# Supplementary material for: Estimated total cardiovascular risk in a rural area of Bangladesh: a household level cross-sectional survey done by local community health workers
Source: BMJ Open. 2021 Aug 4;11(8):e046195. doi: 10.1136/bmjopen-2020-046195 (PMC8340298; doi:10.1136/bmjopen-2020-046195)
Supplement: Author's manuscript [file bmjopen-2020-046195.draft_revisions.pdf]

BMJ Open is committed to open peer review. As part of this commitment we make the peer review history of every article we publish publicly available.

When an article is published we post the peer reviewers' comments and the authors' responses online. We also post the versions of the paper that were used during peer review. These are the versions that the peer review comments apply to.

The versions of the paper that follow are the versions that were submitted during the peer review process. They are not the versions of record or the final published versions. They should not be cited or distributed as the published version of this manuscript.

BMJ Open is an open access journal and the full, final, typeset and author-corrected version of record of the manuscript is available on our site with no access controls, subscription charges or pay-per-view fees (http://bmjopen.bmj.com).

If you have any questions on BMJ Open's open peer review process please email info.bmjopen@bmj.com

BMJ Open

Estimated total cardiovascular risk in a rural area of Bangladesh: A household level cross-sectional survey done by local community health workers

Journal:	*BMJ Open*
Manuscript ID	bmjopen-2020-046195
Article Type:	Original research
Date Submitted by the Author:	26-Oct-2020

Complete List of Authors:	Zaman, MM; World Health Organization Regional Office for South-East Asia, WHO Country Office for Bangladesh<br>Moniruzzaman, Mohammad; Shiga University of Medical Science, Public Health<br>Chowdhury, Kamrun ; National Centre for Control of Rheumatic Fever and Heart Diseases, Epidemiology<br>Zareen, Salma; National Centre for Control of Rheumatic Fever and Heart Diseases, Director<br>Hossain, A.H.M. Enayet; Directorate General of Health Services, NonCommunicable Diseases Control Programme

Keywords:	Cardiac Epidemiology < CARDIOLOGY, PREVENTIVE MEDICINE, PRIMARY CARE, Hypertension < CARDIOLOGY, DIABETES & ENDOCRINOLOGY

SCHOLARONE™
Manuscripts

I, the Submitting Author has the right to grant and does grant on behalf of all authors of the Work (as defined in the below author licence), an exclusive licence and/or a non-exclusive licence for contributions from authors who are: i) UK Crown employees; ii) where BMJ has agreed a CC-BY licence shall apply, and/or iii) in accordance with the terms applicable for US Federal Government officers or employees acting as part of their official duties; on a worldwide, perpetual, irrevocable, royalty-free basis to BMJ Publishing Group Ltd ("BMJ") its licensees and where the relevant Journal is co-owned by BMJ to the co-owners of the Journal, to publish the Work in this journal and any other BMJ products and to exploit all rights, as set out in our licence.

The Submitting Author accepts and understands that any supply made under these terms is made by BMJ to the Submitting Author unless you are acting as an employee on behalf of your employer or a postgraduate student of an affiliated institution which is paying any applicable article publishing charge ("APC") for Open Access articles. Where the Submitting Author wishes to make the Work available on an Open Access basis (and intends to pay the relevant APC), the terms of reuse of such Open Access shall be governed by a Creative Commons licence – details of these licences and which Creative Commons licence will apply to this Work are set out in our licence referred to above.

Other than as permitted in any relevant BMJ Author's Self Archiving Policies, I confirm this Work has not been accepted for publication elsewhere, is not being considered for publication elsewhere and does not duplicate material already published. I confirm all authors consent to publication of this Work and authorise the granting of this licence.

For peer review only

Estimated total cardiovascular risk in a rural area of Bangladesh: A household level cross-sectional survey done by local community health workers

Running title: Ten-year CVD risk in a rural community of Bangladesh

Mohammad Mostafa Zaman[1], Mohammad Moniruzzaman [2], Kamrun Nahar Chowdhury[3], Salma Zareen[3], AHM Enayet Hussain[4]

Author Affiliations:
[1]World Health Organization, Country Office for Bangladesh, Dhaka, Bangladesh; email: zamanm@who.int
[2]Center for Epidemiologic Research in Asia, Shiga University of Medical Science, Otsu, Shiga, Japan; email: mmzbd82@gmail.com
[3] National Center for Control of Rheumatic Fever and Heart Diseases, Dhaka, Bangladesh; email: c.kamrun2014@gmail.com, s.zareen@live.com
[4]Directorate General of Health Services, Dhaka, Bangladesh; email: paedeye@yahoo.com

ORCID:
Mohammad Mostafa Zaman (000-0002-1736-1342);
Mohammad Moniruzzaman (0000-0003-2144-7111);
Kamrun Nahar Chowdhury (0000-0001-9465-9655);

Word counts: Abstract: 285; Main text: 2802

Number of tables: 3

Number of figures: 1

Address for correspondence:

Dr M Mostafa Zaman
Adviser Research and Publication
World Health Organization
10 Gulshan Avenue, Dhaka 1212
Bangladesh
Phone: +88 0960 40 27200 (hunting)
Email: zamanm@who.int

For peer review only - http://bmjopen.bmj.com/site/about/guidelines.xhtml

Abstract

Objective: The aim of this study was to estimate 10-year cardiovascular disease (CVD) risk among Bangladeshi rural community residents, using the 2014 World Health Organization/International Society of Hypertension (WHO/ISH) Risk Prediction Charts.

Study design: Cross-sectional population-based study done by local community health care workers engaging the lowest level facilities of the primary health care system.

Setting and participants: A total of 1545 rural adults aged ≥40 years of Dehatta Upazila of Satkhira district of Bangladesh participated in this survey done in 2015. The community health workers collected data on age, smoking, blood pressure, blood glucose, blood pressure, and treatment history of diabetes and hypertension.

Primary outcome measures: We estimated total 10-year CVD risk using the WHO/ISH South East Asia Region-D charts without cholesterol and categorized the risk into low (<10%), moderate (10 to 19.9%), high (20 to 29.9%), and very high (≥30%).

Results: The participants' mean age (±SD) was 53.9±11.6 years. Overall, the a 10-year CVD risk (%, 95% CI) were as follows: low risk (81.55%, 78.4-84.6), moderate risk (9.9, 7.4–12.1), high risk (5.8, 4.4–7.2), and very high risk (2.8, 1.5–4.1). In women, moderate to very high risks were higher (moderate 12.1%, high 6.1%, and very high 3.7%) compared to men (moderate 7.5%, high 5.5%, and very high 1.9%) but none of these were statistically significant. The very high risk increased from 2.8% to 13.1% when blood pressure ≥160/100 mmHg added. Further addition to medication for hypertension treatment increased it to 21.2%.

Conclusion: The very high-risk estimates could be used for planning resource for CVD prevention programme at Upazila level. There is a need for a national level study, covering diversities of rural areas, to contribute to national planning of CVD prevention.

Keywords: Rural community, cardiovascular disease, cardiovascular risk, Bangladesh

For peer review only

Article Summary

Strengths and limitations of the study

- The present study is the first-ever population-based study in Bangladesh done by community health workers to identify those who need drug therapy CVD prevention (using the 2014 WHO/ISH CVD risk prediction charts) under guidance of the local health authority.
- The use of local community health workers to conduct the survey at household level provided an inherent strength of community participation. Current study has a high potential of replication all over Bangladesh because Upazila health system is similar everywhere.
- The estimates that we present here are based on South-East Asian regional criteria of the CVD Risk Charts in the absence of any national criteria is an inherent weakness of the approach.
- There is threat of generalizability because the survey was done in a single Upazila of a country having hundreds of Upazila.
- Absence of cholesterol data and use of casual blood glucose data in absence of classic symptoms of diabetes are limitations too.

For peer review only

For peer review only - http://bmjopen.bmj.com/site/about/guidelines.xhtml

BACKGROUND

Cardiovascular disease (CVD) is the leading cause of deaths globally, taking an estimated 17.9 million lives in 2016, representing 31% of all deaths [1, 2]. Of these deaths, 85% are due to heart attack and stroke. Over three quarters of CVD deaths take place in low-and middle-income countries (LMICs) [3]. The World Health Organization's (WHO) South-East Asia Region has eleven countries, all of which fall under the LMIC category.

Deaths from CVDs are, to a great extent, preventable through targeting their risk factors. These risk factors include smoking, unhealthy diet, alcohol in excess, sedentary behavior, high blood glucose and high blood pressure. Identification and management of individual risk factors need a huge effort from the health system. A total risk approach developed by WHO/ISH makes the approach easier and cost-effective. [2]

In Bangladesh, a South-East Asian country, 30% of the total deaths were due to CVD in 2016 [2]. National health system's response to identify population at high risk for CVDs is still absent [2]. The availability of trained health workforce, especially community health workers, is a prerequisite to implementing such programmes. Primary health care system in rural areas of Bangladesh includes fixed facility services (Upazila health complex, union sub-centres and community clinics), and domiciliary services provided by community health workers. The community clinics (total 13,000) are the lowest level and most peripheral facilities catering services to approximately 6,000 people of surrounding area.[4] These clinics are run by community health care providers for catering services for selected communicable diseases [4]. They screen patients for hypertension and diabetes and treat them under the guidance of doctors working in the union sub-centres and upazila health complexes. However, their linkage to the community health workers are not well established.

Our previous study showed that the basic Package for Essential NCD (PEN) intervention for NCD prevention, especially CVD, is a feasible and realistic option at primary health care level [5]. However, the proportion of rural people who are at high risk of CVDs are not known. The community health workers' capacity to contribute to CVD prevention at community level is also unknown. Their engagement in a low resource setting like ours might substantially contribute to primary prevention efforts for screening, counseling and community awareness.

For peer review only - http://bmjopen.bmj.com/site/about/guidelines.xhtml

Therefore, we aimed at conducting a household level assessment by community health workers to determine the proportion of population at high-risk for CVD in the catchment area of community clinics of a selected upazila in Bangladesh.

METHODS

Study design and setting

Bangladesh has 492 Upazila (as of 19 December 2017), which is the second lowest tier of regional administration in Bangladesh after a district (total 64). One upazila on average has eight unions. Debhata is one of the Upazila of Satkhira district located in south-western part of Bangladesh. It's projected population for 2015 was 132,303. The adults of Debhatta have five years of median schooling [5], which is classical of Bangladeshi rural area. We selected Debhata upazila because the country's first NCD intervention was implemented there in 2013-2014 [5].

We obtained ethical approval for this study from the ethics committee of the National Center for Control of Rheumatic Fever and Heart Diseases. We provided participants with detailed information regarding the study objectives, procedures and how the data will be utilized by the local health authority using a print and verbal means in Bangla. We informed potential participants that they deserve the right not to participate and withdraw from the study at any stage of data collection. Consent was obtained verbally considering a high illiteracy rate in the area as approved by the ethics committee.

We analyzed data from a household-based assessment done by community health workers of Debhatta upazila from August to December 2015. Data were collected from those aged 40 years or older residing in the catchment area of these 16 community clinics. These 16 community clinics were located in 16 of 57 "Mauza" (the primary sampling units of rural area designated by the statistical authority of Bangladesh). All these community clinics had a list of households in their respective "Mauza". In each Mauza, community health workers selected 100 eligible residents consecutively starting from the first household in the list and continued visiting households until 100 could be selected. Thus, the total sample size was 1600. At least two home visits were done to keep the non-response minimum. As usual, several people were not present at home during data collectors' visit. Refusal was almost absent. Before deployment for the data collection, 16 community health workers from their respective area were trained for three days.

For peer review only - http://bmjopen.bmj.com/site/about/guidelines.xhtml

They were given a set of equipment: analogue weighing scale, flexible metallic tape, aneroid sphygmomanometer, stethoscope, and glucometer (Accu-chek Advantage). A supervised dry-run of the data collection was done on the fourth day.

Patient and Public Engagement

There was no patient involvement.

Data Collection

The community health workers interviewed subjects, measured height, weight and causal capillary blood glucose. The interview included information on age, sex, education, occupation, tobacco use, diet (salt, vegetable and fruit), physical activity, treatment for hypertension and diabetes, and history of CVD event. The questionnaire was in Bangla, adapted from the national STEPS survey 2010 [6]. Blood pressure was measured twice using an appropriately sized arm cuff while the participants were in seating position. The first measurement was performed after five minutes of rest, and the second measurement was taken two minutes after the first measurement. We used the second measurement values in the analyses. Capillary blood glucose irrespective of prandial status [7] was measured using a glucometer on the right index finger avoiding much stasis.

Estimation of 10-year CVD risk

We estimated 10-year CVD risk using 2014 WHO/ISH risk prediction charts (hereinafter referred to as CVD risk chart) for South East Asia Region-D without cholesterol [8, 9] for a fatal or non-fatal major cardiovascular event. The variables used for this estimation were age (40–49 years, 50-59 years, 60-69 years, and ≥70 years), sex (men and women), systolic blood pressure (<140, 140-159, 160-179, and ≥180 mmHg), smoking status (smoker or non-smoker), and the presence or absence of diabetes (casual capillary blood glucose >11.1 mmol/L or use of medication for diabetes). The 10-year CVD risk categories were <10%, 10 – 19.9%, 20-29.9%, and ≥ 30%.

Data Analysis

For peer review only

A total of 1,600 participants (734 were men and 866 women) could be recruited by approaching 1,721 consecutive eligible subjects (response rate, 93.0%). Among them, 55 had a history of CVD events (heart attack or stroke) and were excluded from our analysis as recommended [8]. Finally, our analysis is based on 1545 persons.

Mean (standard deviation) for continuous variables and proportion for categorical variables were obtained. Results on risk categories were presented for sexes combined and separately. Age stratification of the risk categories were not done because it has already been taken in to account for calculating the risk. We categorized the CVD risk into low (<10%), moderate (10 to 19.9%), high (20 to 29.9%), and very high (≥30%) [8]. Prevalence estimates and 95% confidence intervals (CIs) were weighted [10] to account for selection probabilities and non-response to remove bias from a survey sample and make the results better project the target population (all citizens of Debhatta aged 40 years or older). The 95% CIs of the risk estimates were used to examine differences between groups, overlapping intervals were considered non-significant different at 5% level.

Besides, we checked whether the distribution of some established risk factors that were not used in the risk estimations (e.g., low fruit and vegetable intake, sedentary behavior, and obesity) varied among estimated risk categories. Furthermore, we have estimated the prevalence after adding those with blood pressure ≥160/100 mm Hg to the chart-calculated proportions as per CVD risk chart practice notes for clinicians [8, 11]. The notes state that those who have blood pressure ≥160/100 mmHg should be considered in the high-risk category regardless of the risk estimates obtained using the chart. Further categories were created, adding those who had a history of anti-hypertensive medication [11].

RESULTS

<u>Background Characteristics</u>

Results are based on 697 men (45.1%) and 848 women (54.9%) (**Table 1**). Their mean age was 53.9 years, with a standard deviation of 11.6 years. The number of people persistently decreased from (42.4%) in the youngest age group (40-49 years) to 13.9% in the oldest group (70 years or older). Less than one-quarter (24.3%) of them completed primary education. The majority (86.1%) of women were housewives, and the most common occupation in men was agriculture and other manual works (45.8%).

For peer review only - http://bmjopen.bmj.com/site/about/guidelines.xhtml

Smoking was almost absent in women (1.2%) but common in men (36.6%). Most participants had had SBP< 140 mmHg (76.0%), which gradually declined thru intervening categories to 1.6% in the ≥180 mmHg category. One-third (35.2%) people had hypertension (blood prerssure ≥140/90 mmHg or history of medication), and 9.4% had diabetes (capillary blood glucose >11.1 mmol/L or medication).

Distribution of 10-year CVD Risk

All subsequent analyses were weighted. The distribution of 10-year CVD risk in the study participants are presented for sexes combined and separately (**Table 2**). Most participants had a low risk of CVD (81.5%, 95% CI 78.4–84.6), while the moderate, high, and very high risk were 9.9 % (7.4–12.1), 5.8% (4.4–7.2), and 2.8% (1.5–4.1) respectively. In general, women had relatively high prevalence of moderate, high, and very high risk compared to men, albeit statistically non-significant as indicated by overlapping 95% CIs.

We examined whether some other potential risk factors had influenced the 10-year CVD risk estimations from low to a very high level. These risk factors included a low intake of fruit and vegetables, sedentary behavior, and obesity (**Figure 1**). There is a general tendency of all these factors except obesity to get higher with higher categories of risk, although these were non-significant as indicated by overlapping 95% CIs.

Irrespective of risk categories, those who have blood pressure≥160/90 mmHg needs treatment. Therefore, we added the proportions of people having blood pressure ≥160/100 mmHg to the ‘very high’ risk categories obtained using the Chart (**Table 3**). This increased the proportion of population with a ‘very high’ CVD risk from 2.8% to 13.1%. With the further addition of self-reported medication history for hypertension, the proportion further increased to 21.2%.

DISCUSSION

The present study is the first-ever population-based study in Bangladesh to estimate the 10-year CVD risk scores done by community health workers. In this household survey of people aged 40 years or older living in a rural area of Bangladesh, we found that 2.8% had very-high CVD risk who require medication for treatment. Addition of very high blood pressure level (≥160/100) and history of medication for hypertension increased this to 13.1% and 21.2% in sequential order.

For peer review only - http://bmjopen.bmj.com/site/about/guidelines.xhtml

These findings underscore the importance of pharmacological interventions in a cost-effective manner and use of community health workers for CVD prevention and control measures.

The Risk Charts were developed for the LMICs [12], which is pertinent for Bangladeshi population. In a representative sample of an Upazila, we found a low risk for the 10-year CVD risk in majority (81.5%) of the adults aged ≥ 40 years living in a rural area. This estimate is comparable to South-East Asia regional estimate [9], and neighboring Asian countries such as Pakistan (79.2%) [12], Nepal (86.4%) [13], Sri Lanka (86.4%) [14], and India (82.7%) [15]. High CVD risk (≥20%) prevalence, on the other hand, in other Asian countries (Cambodia, Malaysia and Mongolia) is also like our finding (1.3% to 6.0%) [11]. These, however, should be interpreted with caution because of differences in age of the subject, which is an important determinant. Another point is the version of the Chart. A few previous studies used 2007 version, we used 2014 version. Therefore, our results may not be directly comparable without considering the version and age group of the subjects. Three studies in Bangladesh reported CVD risk in adults [16-18]. Two of them were done in rural populations. The proportion of people who had high risk (≥ 20%) varied largely from 2.1% [17] to 10.0% [16] between two studies. Therefore, a conclusive prevalence estimate for rural area is yet to be drawn for Bangladesh. There might be community-specific features to influence the risk estimates.

Out of pocket health care expenditure in Bangladesh is very high (60%), and most of this expenditure is for medicines [19]. We believe that the use of 10-year CVD risk would decrease the out of pocket- and other-costs [12]. The total risk approach may help reducing the patients' load to the health system too, which is already overburdened in Bangladesh. Because the community health workers can detect CVD risk at household level, the coverage of primary health care for the prevention of CVD events would be more efficient if they are used. The prevention of catastrophic CVD events can reduce impoverishment linked to CVD events [20].

In 2016, an estimated 67% of all deaths in Bangladesh were due to NCDs, and the risk of premature death from chronic disease among adults aged 30-70 years was 22% [2]. Despite this substantial burden, preventive services for CVDs in Bangladesh are limited. Only 16% of health care facilities (i.e., hospitals, community clinics) had the resources to diagnose, treat and manage patients with CVDs [21]. The Upazila health complexes provide both preventive and curative services. Next to the Upazila health complexes the union level health and family welfare centres cater services to people through doctors, medical assistants and family welfare visitors

For peer review only - http://bmjopen.bmj.com/site/about/guidelines.xhtml

depending on their availability. The ultimate level of health care facilities in rural areas of Bangladesh are the community clinics at the door steps of people. The services at these facilities are limited to the measurement of blood pressure or referrals only [21]. These centres can provide better services if these can be integrated into the domiciliary services provided by the community health workers. The current study provides evidences, for the first time, that health workers can screen out potential CVD risk for further evaluation at next level.

Although we provide evidence that community health care workers can contribute to detection of people with a high risk of CVD for initiating treatment at primary health care facilities by physicians, they need to be trained well. Further studies are warranted to study more on the cut-off points (20% or 30%) of CVD risk charts for initiating medicines. Both these cut-off points have potentials of over-and -under-diagnosis. Nonetheless, most studies favor a 30% cut-off point for CVD risk estimation. WHO has recommended that 80% of health facilities should have the availability of affordable basic technologies and essential medicines necessary to treat significant NCDs, including CVDs [22]. Screening of rural adults by community health workers could contribute substantially to achieve this target.

The number of people who needs these services based on single risk factor would be huge to deal by a limited number of health service providers where supplies and medicines remain scarce. By integrating the CVD risk prediction charts into the national guidelines for the management of NCDs, the resource needs could be easily reduced without compromising the quality of services to those who are at high risk of CVDs. The implementation of CVD risk prediction chart may become popular if adequate supply of essential medicines is ensured, which is currently inadequate [5], to fixed facilities where they will be referred. The use of local health assistants to conduct the survey at household level provided an inherent strength of community participation. Considering the similarity of the environment around the community clinics the current study has a high potential of replication all over Bangladesh.

Our study had limitations too. The estimates that we present here are based on South-East Asian regional criteria of the CVD Risk Charts in the absence of any national criteria. There might be between-country difference in applicability of the prediction charts. Besides, the study was done in a single upazila, not in Bangladesh as a whole. Absence of cholesterol data and use of casual blood glucose data in absence of classic symptoms of diabetes are limitations too. Further, our data were collected back in 2015, and we cannot analyze out data using 2019 CVD

For peer review only - http://bmjopen.bmj.com/site/about/guidelines.xhtml

risk chart [9] because the control measures were already planned based on our findings. The 2019 CVD risk chart considered obesity for calculating the risk. We presume that the data of CVD risk using the 2019 charts will be different than what we report here [23, 24].

Conclusion

We conclude that the implementation of a total risk approach for preventing CVDs engaging community health workers and community clinics is a feasible option in Bangladesh. This approach has a high potential for better coverage of essential health care and prevention of premature CVDs among those who have a very high risk of CVD events. There is a need for national level study covering diversities of rural areas. Future studies should consider including the obesity and total cholesterol in line with 2019 CVD prediction chart.

Footnotes

Ethical considerations

This survey was done by the Upazila Health System to see whether they community workers can get CVD risk factor data and provide feedbacks to the community. In addition, acceptance of household level measurement of blood pressure etc and counselling services provided by the community health workers were examined. Communities were encouraged to participate. Community consents were obtained engaging the management committee of the community clinics, union councils and management committees of all schools before launching the initiative. These were in line with the ethics committee approval of the National Center for Control of Rheumatic Fever and Heart Diseases. The Committee approved getting the consent verbally because this assessment was a health service-oriented work as per the mandate of the Upazila Health Complex. Participants had the rights to withdraw at any stage of the assessment and not to participate at all. Participants were given a report of their risk factor status and referral was done for treatment if necessary. All the health workers of the Upazila irrespective of their involvement in the assessment were trained so that the health benefits reach out all the households of Debhatta Upazila.

For peer review only - http://bmjopen.bmj.com/site/about/guidelines.xhtml

Consent to publish: All authors have given consent to the publication of this manuscript.

Competing Interests: The authors of this study declare no conflict of interest.

Funding: Financial assistance for this study was provided by the WHO Bangladesh (WHO Reference: 2015/545398-0, Purchase Order: 201288322)

Authors contributions: MMZ: conceptualized the project, developed the protocol, trained manpower, analyzed data, interpreted results critically, and drafted the manuscript. MZ, KNC, SZ: prepared the manual and trained the field team, implemented the survey in the field, revised the manuscript, and AHMEH guided the work, coordinated the fieldwork, and reviewed the manuscript critically. He is the guarantor of data. All authors have approved the submission.

Acknowledgments: The study was implemented at upazila level by the field team of the Debhatta Upazila (HI, AHI, and Has) with support from the WHO Bangladesh. The authors are grateful to Upazila Health and Family Planning Officer and his office team for organizing the training etc. Supports were provided by the NCD team of the Directorate General of Health Services.

Disclaimer: The authors alone are responsible for the views expressed in this article, and they do not necessarily represent the views, decisions, or policies of the institutions with which they are affiliated.

Availability of data and materials: The de-identified participant data used and/or analyzed during the current study are available from the corresponding author on reasonable request. Please contact Dr M. Mostafa Zaman at zamanm@who.int for further information and guidelines.

Figure Legend

Figure 1. Weighted prevalence of 10-year CVD risk enhancing factors not included in WHO/ISH risk scores across risk categories* in men and women combined (n=1545)

* figure footnotes:

For peer review only - http://bmjopen.bmj.com/site/about/guidelines.xhtml

a. Using the South East Asia Region-D charts without cholesterol; Low risk, <10%; Moderate risk, 10-19.9%; High risk, 20-29.9%, very high risk, ≥30%;

b. Low vegetable/fruit, <5 servings/day; sedentary behaviour, <600 MET min/week; obesity, body mass index ≥30 kg/metre squared;

For peer review only

For peer review only - http://bmjopen.bmj.com/site/about/guidelines.xhtml

References

1. Kyu HH, Abate D, Abate KH, Abay SM, Abbafati C, Abbasi N, Abbastabar H, Abd-Allah F, Abdela J, Abdelalim A *et al*: **Global, regional, and national disability-adjusted life-years (DALYs) for 359 diseases and injuries and healthy life expectancy (HALE) for 195 countries and territories, 1990–2017: a systematic analysis for the Global Burden of Disease Study 2017**. *The Lancet* 2018; **392**:1859-1922.

2. World Health Oorganization: **Noncommunicable diseases country profiles 2018**. Geneva: World Health Organization; 2018. [accessed on 22 October 2020]; website: https://www.who.int/nmh/publications/ncd-profiles-2018/en/

3. Checkley W, Ghannem H, Irazola V, Kimaiyo S, Levitt NS, Miranda JJ, Niessen L, Prabhakaran D, Rabadan-Diehl C, Ramirez-Zea M *et al*: **Management of NCD in low- and middle-income countries**. *Glob Heart* 2014, **9**:431-443. doi: 10.1016/j.gheart.2014.11.003.

4. Riaz BK, Ali L, Ahmad SA, Islam MZ, Ahmed KR, Hossain S: **Community clinics in Bangladesh: A unique example of public-private partnership**. In: *Heliyon.* vol. 6; 2020: e03950. doi: 10.1016/j.heliyon.2020.e03950.

5. Zaman MM, Ullah AKMJ, Bhuiyan MR, Karim MN, Moniruzzaman M, Rahman SMA. **Noncommunicable disease prevention and control situation in a primary health care setting of Bangladesh: design and baseline findings of an intervention**. *Chronic Dis Int* 2016, **3**:1021. Website: https://www.researchgate.net/publication/307605824_Noncommunicable_Disease_Prevention_and_Control_Situation_in_a_Primary_Health_Care_Setting_of_Bangladesh_Design_and_Baseline_Findings_of_an_Intervention

6. Zaman MM, Rahman MM, Rahman MR, Bhuiyan MR, Karim MN, Chowdhury MA: **Prevalence of risk factors for non-communicable diseases in Bangladesh: Results from STEPS survey 2010**. *Indian J Public Health* 2016, **60**:17-25. doi: 10.4103/0019-557X.177290.

7. World Health Organization. **Global report on diabetes 2016. Geneva, Switzerland.** [https://www.who.int/diabetes/global-report/en/]

8. **World Health Organization/ International Society of Hypertension (WHO/ISH) risk prediction charts** for 14 WHO epidemiological sub-regions (charts in colour) https://www.who.int/ncds/management/WHO_ISH_Risk_Prediction_Charts.pdf?ua=1

9. The WHO CVD Risk Chart Working Group. **World Health Organization cardiovascular disease risk charts: revised models to estimate risk in 21 global regions**. The *Lancet Glob Health* 2019, **7**:e1332-e1345. DOI:https://doi.org/10.1016/S2214-109X(19)30318-3

For peer review only - http://bmjopen.bmj.com/site/about/guidelines.xhtml

10. Hakim F, Bhuiyan R, Akter K, Zaman M. **Weighting National Survey Data for Bangladeshi Population**. In: SAGE Research Methods Cases: Medicine and Health, London; 2020. DOI**:** https://dx.doi.org/10.4135/9781529743623

11. Otgontuya D, Oum S, Buckley BS, Bonita R: **Assessment of total cardiovascular risk using WHO/ISH risk prediction charts in three low and middle income countries in Asia**. *BMC Public Health* 2013;**13**:539. doi: 10.1186/1471-2458-13-539.

12. Mendis S, Lindholm LH, Anderson SG, Alwan A, Koju R, Onwubere BJ, Kayani AM, Abeysinghe N, Duneas A, Tabagari S *et al*: **Total cardiovascular risk approach to improve efficiency of cardiovascular prevention in resource constrain settings**. *J Clin Epidemiol* 2011;**64**:1451-1462. doi: 10.1016/j.jclinepi.2011.02.001. Epub 2011 May 6.

13. Khanal MK, Ahmed MS, Moniruzzaman M, Banik PC, Dhungana RR, Bhandari P, Devkota S, Shayami A: **Total cardiovascular risk for next 10 years among rural population of Nepal using WHO/ISH risk prediction chart**. *BMC Res Notes* 2017, **10**:120. doi: 10.1186/s13104-017-2436-9.

14. Ranawaka UK, Wijekoon CN, Pathmeswaran A, Kasturiratne A, Gunasekera D, Chackrewarthy S, Kato N, Wickramasinghe AR: **Risk estimates of cardiovascular diseases in a Sri Lankan community**. *Ceylon Med J* 2016, **61**:11-17. doi: 10.4038/cmj.v61i1.8253.

15. Ghorpade AG, Shrivastava SR, Kar SS, Sarkar S, Majgi SM, Roy G: **Estimation of the cardiovascular risk using World Health Organization/International Society of Hypertension (WHO/ISH) risk prediction charts in a rural population of South India**. *Int J Health Policy Manag* 2015;**4**:531-536. doi: 10.15171/ijhpm.2015.88.

16. Fatema K, Zwar NA, Milton AH, Rahman B, Ali L: **Application of two versions of the WHO/international society of hypertension absolute cardiovascular risk assessment tools in a rural Bangladeshi population**. *BMJ Open* 2015, **5**:e008140. doi: 10.1136/bmjopen-2015-008140.

17. Cravedi P, Sharma SK, Bravo RF, Islam N, Tchokhonelidze I, Ghimire M, Pahari B, Thapa S, Basnet A, Tataradze A *et al*: **Preventing renal and cardiovascular risk by renal function assessment: insights from a cross-sectional study in low-income countries and the USA**. *BMJ Open* 2012;2: e001357. doi: 10.1136/bmjopen-2012-001357.

18. Ahmed MSAM, Moniruzzaman M, Chowdhury S, Banik PC. **Cardiovascular Risk Assessment Among Urban Population of Bangladesh Using WHO/ISH Risk Prediction Chart**. *International Journal of Epidemiology* 2015, **44**(suppl_1):i202-i202.

19. Government of People's Republic of Bangladesh. **Multisectoral Action Plan for the Noncommunicable Disease Control and Prevention 2018-205 with a three-year operational plan**. [Accessed: 22 October 2020] https://dghs.gov.bd/images/docs/Publicaations/NCDC_multisectoral_action_plan_2018_2025.pdf

For peer review only - http://bmjopen.bmj.com/site/about/guidelines.xhtml

20. Bangladesh Bureau of Statistics, Government of People's Republic of Bangladesh. **Health and Morbidity Status Survey-2012.** Dhaka, December 2013. [Accessed 22 October 2020] http://bbs.portal.gov.bd/sites/default/files/files/bbs.portal.gov.bd/page/4c7eb0f0_e780_4686_b546_b4fa0a8889a5/HMSS.pdf

21. National Institute of Population Research and Training (NIPORT), Associates for Community and Population Research (ACPR), and ICF International. 2016. Bangladesh Health Facility Survey 2014. Dhaka, Bangladesh: NIPORT, ACPR, and ICF International. **Bangladesh Health Facility Survey 2014.** [Accessed 22 October 2020]. [https://dhsprogram.com/pubs/pdf/SPA23/SPA23.pdf]

22. World Health Organization. **NCD Global Monitoring Framework: Ensuring progress on noncommunicable diseases in countries. Geneva: World Health Organization.** [Accessed: 22 October 2020] https://www.who.int/nmh/global_monitoring_framework/2013-11-06-who-dc-c268-whp-gap-ncds-techdoc-def3.pdf?ua=1

23. Su TT, Amiri M, Mohd Hairi F, Thangiah N, Bulgiba A, Majid HA. **Prediction of cardiovascular disease risk among low-income urban dwellers in metropolitan Kuala Lumpur, Malaysia**. *Biomed Res Int* 2015;**2015**:516984. doi: 10.1155/2015/516984.

24. Islam JY, Zaman MM, Moniruzzaman M, Shakoor SA, Hossain AHME. **Estimation of total cardiovascular risk using the 2019 WHO CVD prediction charts and comparison of population-level costs based on alternative drug therapy guidelines: a population-based study of adults in Bangladesh.** BMJ Open 2020;10:e035842. doi:10.1136/bmjopen-2019-035842

For peer review only

For peer review only - http://bmjopen.bmj.com/site/about/guidelines.xhtml

Table 1. Characteristics of the study participants, Bangladeshi rural adults aged 40 years or older years, results are number (percent)

Characteristics	Total (n = 1545)	Men (n = 697)	Women (n = 848)
Age categories			
40-49	655 (42.4)	229 (32.9)	426 (50.2)
50-59	425 (27.5)	209 30.0	216 (25.4)
60-69	250 (16.2)	130 (18.7)	120 (14.2)
≥70	215 (13.9)	129 (18.5)	86 (10.1)
Education, above primary	375 (24.3)	247 (35.4)	128 (15.1)
Occupation			
Business (small or big)	232 (15.0)	228 (32.4)	4 (0.5)
Agriculture and other manual works	358 (23.2)	322 (45.8)	36 (4.3)
Household works	749 (48.4)	21 (3.0)	728 (86.1)
Others¶	207 (13.4)	132 (18.8)	75 (8.9)
Smoking	265 (17.2)	255 (36.6)	10 (1.2)
Systolic blood pressure, mmHg			
<140	1174 (76.0)	571 (81.9)	603 (71.1)
140-159	257 (16.6)	95 (13.6)	162 (19.1)
160-179	85 (5.5)	23 (3.3)	61 (7.3)
≥180	27 (1.8)	6 (0.9)	21 (2.5)
Diastolic blood pressure, mmHg			
<90	1109 (71.8)	528 (75.6)	581 (68.5)
90-99	263 (17.2)	113 (16.2)	150 (17.7)
≥100	173 (11.2)	56 (8.0)	117 (13.8)
Hypertension§	556 (36.0)	206 (29.6)	350 (41.3)
Diabetes†	141 (9.1)	65 (9.3)	76 (9.0)

¶Includes: Salaried work, unemployed, weaver, beggar, cook, tailor, cobbler, etc.
§Blood pressure≥140/90 mmHg, or use of antihypertension medication;
†Random capillary glucose ≥11.1 mmol/L, or use of anti-diabetes medication

For peer review only - http://bmjopen.bmj.com/site/about/guidelines.xhtml

Table 2. Percent (95% confidence interval) people having categories of 10-year CVD risk (fatal and non-fatal) *

CVD risk categories	Men and women (n=1545)	Men (n=697)	Women (n=848)
<10 %	81.5 (78.4-84.6)	85.0 (80.4-89.6)	78.2 (74.4-81.9)
10-19.9%	9.9 (7.4-12.1)	7.5 (5.2-9.9)	12.1 (9.0-15.2)
20-29.9%	5.8 (4.4-7.2)	5.5 (3.4-7.6)	6.1 (4.5-7.6)
≥30%	2.8 (1.5-4.1)	1.9 (0.4-2.5)	3.7 (1.9-5.5)

*Using the WHO/ISH South East Asia Region-D charts without cholesterol;

467

468

For peer review only

Table 3. Prevalence (95% confidence interval) of people aged ≥40 years with CVD risk ≥30% plus BP* ≥160/100 plus medication for hypertension

Chart and/or hypertension	Men and women (n=1545)	Men (n=697)	Women (n=848)
Simple use of chart	2.8 (1.5-4.1)	1.9 (0.4-3.5)	3.7 (1.9-5.5)
Chart plus BP≥160/100 mmHg	13.1 (9.8-16.5)	8.9 (5.2-12.6)	17.1 (13.1-21.1)
Chart plus BP≥160/100 mmHg plus hypertension medication	21.2 (16.5-26.0)	15.5 (9.6-21.4)	26.6 (20.9-32.2)

*BP indicates blood pressure

469

470

For peer review only

Figure legend

Figure 1. Weighted prevalence of 10-year CVD risk enhancing factors not included in WHO/ISH risk scores across risk categories* in men and women combined (n=1545)

*footnote: Using the South East Asia Region-D charts without cholesterol; Low risk, <10%; Moderate risk, 10-19.9%; High risk, 20-29.9%, very high risk, ≥30%; Low vegetable/fruit intake, <5 servings/day, sedentary behaviour, <600 MET min/week; obesity, body mass index ≥30 kg/m^2

For peer review only

For peer review only - http://bmjopen.bmj.com/site/about/guidelines.xhtml

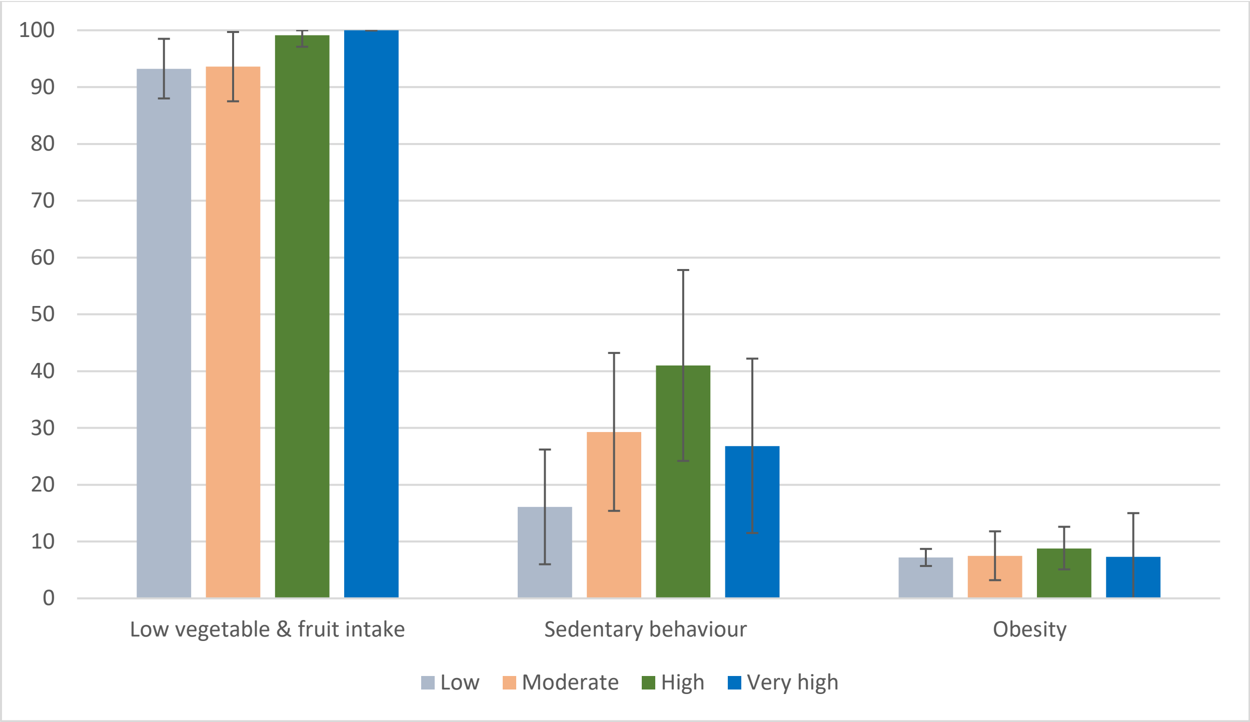
100
90
80
70
60
50
40
30
20
10
0
Low vegetable & fruit intake
Sedentary behaviour
Obesity
Low
Moderate
High
Very high

STROBE Statement—Checklist of items that should be included in reports of ***cross-sectional studies***

	Item No	Recommendation	Page No
**Title and abstract**	1	(*a*) Indicate the study's design with a commonly used term in the title or the abstract	1
		(*b*) Provide in the abstract an informative and balanced summary of what was done and what was found	2
**Introduction**			
Background/rationale	2	Explain the scientific background and rationale for the investigation being reported	4
Objectives	3	State specific objectives, including any prespecified hypotheses	5
**Methods**			
Study design	4	Present key elements of study design early in the paper	5
Setting	5	Describe the setting, locations, and relevant dates, including periods of recruitment, exposure, follow-up, and data collection	5
Participants	6	(*a*) Give the eligibility criteria, and the sources and methods of selection of participants	5
Variables	7	Clearly define all outcomes, exposures, predictors, potential confounders, and effect modifiers. Give diagnostic criteria, if applicable	6
Data sources/ measurement	8*	For each variable of interest, give sources of data and details of methods of assessment (measurement). Describe comparability of assessment methods if there is more than one group	*6-7*
Bias	9	Describe any efforts to address potential sources of bias	6
Study size	10	Explain how the study size was arrived at	4-5
Quantitative variables	11	Explain how quantitative variables were handled in the analyses. If applicable, describe which groupings were chosen and why	6-7
Statistical methods	12	(*a*) Describe all statistical methods, including those used to control for confounding	6-7
		(*b*) Describe any methods used to examine subgroups and interactions	6-7
		(*c*) Explain how missing data were addressed	6
		(*d*) If applicable, describe analytical methods taking account of sampling strategy	na
		(*e*) Describe any sensitivity analyses	na
**Results**			
Participants	13*	(a) Report numbers of individuals at each stage of study—eg numbers potentially eligible, examined for eligibility, confirmed eligible, included in the study, completing follow-up, and analysed	6-7
		(b) Give reasons for non-participation at each stage	6
		(c) Consider use of a flow diagram	na
Descriptive data	14*	(a) Give characteristics of study participants (eg demographic, clinical, social) and information on exposures and potential confounders	7-8
		(b) Indicate number of participants with missing data for each variable of interest	na
Outcome data	15*	Report numbers of outcome events or summary measures	6-7
Main results	16	(*a*) Give unadjusted estimates and, if applicable, confounder-adjusted estimates and their precision (eg, 95% confidence interval). Make clear which confounders were adjusted for and why they were included	7-8

For peer review only - http://bmjopen.bmj.com/site/about/guidelines.xhtml

		(*b*) Report category boundaries when continuous variables were categorized	6
		(*c*) If relevant, consider translating estimates of relative risk into absolute risk for a meaningful time period	na
Other analyses	17	Report other analyses done—eg analyses of subgroups and interactions, and sensitivity analyses	na
**Discussion**			
Key results	18	Summarise key results with reference to study objectives	8
Limitations	19	Discuss limitations of the study, taking into account sources of potential bias or imprecision. Discuss both direction and magnitude of any potential bias	10
Interpretation	20	Give a cautious overall interpretation of results considering objectives, limitations, multiplicity of analyses, results from similar studies, and other relevant evidence	10
Generalisability	21	Discuss the generalisability (external validity) of the study results	10
**Other information**			
Funding	22	Give the source of funding and the role of the funders for the present study and, if applicable, for the original study on which the present article is based	11

*Give information separately for exposed and unexposed groups.

Note: An Explanation and Elaboration article discusses each checklist item and gives methodological background and published examples of transparent reporting. The STROBE checklist is best used in conjunction with this article (freely available on the Web sites of PLoS Medicine at http://www.plosmedicine.org/, Annals of Internal Medicine at http://www.annals.org/, and Epidemiology at http://www.epidem.com/). Information on the STROBE Initiative is available at www.strobe-statement.org.

For peer review only

BMJ Open

Estimated total cardiovascular risk in a rural area of Bangladesh: A household level cross-sectional survey done by local community health workers

Journal:	*BMJ Open*
Manuscript ID	bmjopen-2020-046195.R1
Article Type:	Original research
Date Submitted by the Author:	01-Feb-2021
Complete List of Authors:	Zaman, MM; World Health Organization Regional Office for South-East Asia, WHO Country Office for Bangladesh<br>Moniruzzaman, Mohammad; Shiga University of Medical Science, Public Health<br>Chowdhury, Kamrun ; National Centre for Control of Rheumatic Fever and Heart Diseases, Epidemiology<br>Zareen, Salma; National Centre for Control of Rheumatic Fever and Heart Diseases, Director<br>Hossain, A.H.M. Enayet; Directorate General of Health Services, NonCommunicable Diseases Control Programme
**Primary Subject Heading**:	Cardiovascular medicine
Secondary Subject Heading:	Public health
Keywords:	Cardiac Epidemiology < CARDIOLOGY, PREVENTIVE MEDICINE, PRIMARY CARE, Hypertension < CARDIOLOGY, DIABETES & ENDOCRINOLOGY

SCHOLARONE™
Manuscripts

I, the Submitting Author has the right to grant and does grant on behalf of all authors of the Work (as defined in the below author licence), an exclusive licence and/or a non-exclusive licence for contributions from authors who are: i) UK Crown employees; ii) where BMJ has agreed a CC-BY licence shall apply, and/or iii) in accordance with the terms applicable for US Federal Government officers or employees acting as part of their official duties; on a worldwide, perpetual, irrevocable, royalty-free basis to BMJ Publishing Group Ltd ("BMJ") its licensees and where the relevant Journal is co-owned by BMJ to the co-owners of the Journal, to publish the Work in this journal and any other BMJ products and to exploit all rights, as set out in our licence.

The Submitting Author accepts and understands that any supply made under these terms is made by BMJ to the Submitting Author unless you are acting as an employee on behalf of your employer or a postgraduate student of an affiliated institution which is paying any applicable article publishing charge ("APC") for Open Access articles. Where the Submitting Author wishes to make the Work available on an Open Access basis (and intends to pay the relevant APC), the terms of reuse of such Open Access shall be governed by a Creative Commons licence – details of these licences and which Creative Commons licence will apply to this Work are set out in our licence referred to above.

Other than as permitted in any relevant BMJ Author's Self Archiving Policies, I confirm this Work has not been accepted for publication elsewhere, is not being considered for publication elsewhere and does not duplicate material already published. I confirm all authors consent to publication of this Work and authorise the granting of this licence.

For peer review only

Estimated total cardiovascular risk in a rural area of Bangladesh: A household level cross-sectional survey done by local community health workers

Running title: Ten-year CVD risk in a rural community of Bangladesh

Mohammad Mostafa Zaman[1], Mohammad Moniruzzaman [2], Kamrun Nahar Chowdhury[3], Salma Zareen[3], AHM Enayet Hussain[4]

Author Affiliations:
[1]World Health Organization, Country Office for Bangladesh, Dhaka, Bangladesh; email: zamanm@who.int
[2]Center for Epidemiologic Research in Asia, Shiga University of Medical Science, Otsu, Shiga, Japan; email: mmzbd82@gmail.com

[3] National Center for Control of Rheumatic Fever and Heart Diseases, Dhaka, Bangladesh; email: c.kamrun2014@gmail.com, s.zareen@live.com

[4]Directorate General of Health Services, Dhaka, Bangladesh; email: paedeye@yahoo.com

ORCID:
Mohammad Mostafa Zaman (000-0002-1736-1342);

Mohammad Moniruzzaman (0000-0003-2144-7111);

Kamrun Nahar Chowdhury (0000-0001-9465-9655);

Word counts: Abstract: 284; Main text: 2796

Number of tables: 3

Number of figures: 2

Address for correspondence:

Dr M Mostafa Zaman
Adviser Research and Publication
World Health Organization
10 Gulshan Avenue, Dhaka 1212
Bangladesh
Phone: +88 0960 40 27200 (hunting)
Email: zamanm@who.int

For peer review only

For peer review only - http://bmjopen.bmj.com/site/about/guidelines.xhtml

Abstract

Objective: The aim of this study was to estimate 10-year cardiovascular disease (CVD) risk among Bangladeshi rural community residents, using the 2014 World Health Organization/International Society of Hypertension (WHO/ISH) Risk Prediction Charts.

Study design: Cross-sectional population-based study done by local community health care workers engaging the lowest level facilities of the primary health care system.

Setting and participants: A total of 1545 rural adults aged ≥40 years of Dehatta Upazila of Satkhira district of Bangladesh participated in this survey done in 2015. The community health workers collected data on age, smoking, blood pressure, blood glucose, and treatment history of diabetes and hypertension.

Primary outcome measures: We estimated total 10-year CVD risk using the WHO/ISH South East Asia Region-D charts without cholesterol and categorized the risk into low (<10%), moderate (10 to 19.9%), high (20 to 29.9%), and very high (≥30%).

Results: The participants' mean age (±SD) was 53.9±11.6 years. Overall, the 10-year CVD risks (%, 95% CI) were as follows: low risk (81.55%, 78.4-84.6), moderate risk (9.9%, 7.4–12.1%), high risk (5.8%, 4.4–7.2%), and very high risk (2.8%, 1.5–4.1%). In women, moderate to very high risks were higher (moderate 12.1%, high 6.1%, and very high 3.7%) compared to men (moderate 7.5%, high 5.5%, and very high 1.9%) but none of these were statistically significant. The age-standardized prevalence of very high risk increased from 2.9% (0.7-5.2%) which was increased to 8.5% when those with anti-hypertensive medication having controlled blood pressure (<140/90 mmHg) added.

Conclusion: The very high-risk estimates could be used for planning resource for CVD prevention programme at Upazila level. There is a need for a national level study, covering diversities of rural areas, to contribute to national planning of CVD prevention.

Keywords: Rural community, cardiovascular disease, cardiovascular risk, Bangladesh

For peer review only - http://bmjopen.bmj.com/site/about/guidelines.xhtml

Article Summary

Strengths and limitations of the study

- The present study is the first-ever population-based study in Bangladesh done by community health workers to identify those who need drug therapy CVD prevention (using the 2014 WHO/ISH CVD risk prediction charts) under guidance of the local health authority.
- The use of local community health workers to conduct the survey at household level provided an inherent strength of community participation. Current study has a high potential of replication all over Bangladesh because Upazila health system is similar everywhere.
- The estimates that we present here are based on South-East Asian regional criteria of the CVD Risk Charts in the absence of any national criteria is an inherent weakness of the approach.
- There is threat of generalizability because the survey was done in a single Upazila of a country having hundreds of Upazila.
- Absence of cholesterol data and use of casual blood glucose data in absence of classic symptoms of diabetes are limitations too.

For peer review only

BACKGROUND

Cardiovascular disease (CVD) is the leading cause of deaths globally, taking an estimated 17.9 million lives in 2016, representing 31% of all deaths [1 2]. Of these deaths, 85% are due to heart attack and stroke. Over three quarters of CVD deaths take place in low-and middle-income countries (LMICs) [3]. The World Health Organization's (WHO) South-East Asia Region has eleven countries, all of which fall under the LMIC category.

Deaths from CVDs are, to a great extent, preventable through targeting their risk factors. These risk factors include smoking, unhealthy diet, alcohol in excess, sedentary behavior, high blood glucose and high blood pressure. Identification and management of individual risk factors need a huge effort from the health system. To help reduce the global burden of CVDs, WHO member states have committed to provide counselling and drug treatments for at least 50% of eligible people (defined as aged 40 years or older and at high risk of CVDs) by 2025 [4]. Accordingly, a total risk approach developed by WHO/ISH makes the approach easier and cost-effective. [5 6]

In Bangladesh, a South-East Asian country, 30% of the total deaths were due to CVD in 2016 [2]. National health system's response to identify population at high risk for CVDs is still absent [2]. The availability of trained health workforce, especially community health workers, is a prerequisite to implementing such programmes. Primary health care system in rural areas of Bangladesh includes fixed facility services (Upazila health complex, union sub-centres and community clinics), and domiciliary services provided by community health workers. The community clinics (total 13,000) are the lowest level and most peripheral facilities catering services to approximately 6,000 people of surrounding area.[7] These clinics are run by community health care providers for catering services for selected communicable diseases[7]. They screen patients for hypertension and diabetes and treat them under the guidance of doctors working in the union sub-centres and upazila health complexes. However, their linkage to the community health workers are not well established.

Our previous study showed that the basic Package for Essential NCD (PEN) intervention for noncommunicable disease (NCD) prevention, especially CVD, is a feasible and realistic option at primary health care level [8]. However, the proportion of rural people who are at high risk of CVDs are not known. The community health workers' capacity to contribute to CVD

For peer review only

prevention at community level is also unknown. Their engagement in a low resource setting like ours might substantially contribute to primary prevention efforts for screening, counseling and community awareness. Therefore, we aimed at conducting a household level assessment by community health workers to determine the proportion of population at high-risk for CVD in the catchment area of community clinics of a selected upazila in Bangladesh.

METHODS

Study design and setting

Bangladesh has 492 Upazila (as of 19 December 2017), which is the second lowest tier of regional administration in Bangladesh after a district (total 64). One upazila on average has eight unions. Debhata is one of the Upazila of Satkhira district located in south-western part of Bangladesh. It's projected population for 2015 was 132,303. The adults of Debhatta have five years of median schooling [8], which is classical of Bangladeshi rural area. We selected Debhata upazila because the country's first NCD intervention was implemented there in 2013-2014 [8].

We obtained ethical approval for this study from the ethics committee of the National Center for Control of Rheumatic Fever and Heart Diseases [9]. We provided participants with detailed information regarding the study objectives, procedures and how the data will be utilized by the local health authority using a print and verbal means in Bangla. We informed potential participants that they deserve the right not to participate and withdraw from the study at any stage of data collection. Consent was obtained verbally considering a high illiteracy rate in the area as approved by the ethics committee.

Experienced community health workers collected data at household level from August to December 2015 in Debhatta Upazila. Participants were resident adults aged 40 years or older residing in the catchment areas of all 16 community clinics of the Upazila. These 16 community clinics were located in 16 of 57 "Mauza" (the primary sampling units of rural area designated by the statistical authority of Bangladesh). All these community clinics had a list of households in their respective "Mauza". In each Mauza, community health workers selected 100 eligible residents consecutively starting from the first household in the list and continued visiting households until 100 could be selected. Thus, the total sample size was 1600. At least two home visits were done to keep the non-response minimum. As usual, several people were not present at

For peer review only - http://bmjopen.bmj.com/site/about/guidelines.xhtml

home during data collectors' visit. Refusal was almost absent. Before deployment for the data collection, 16 community health workers from their respective area were trained for three days. They were given a set of equipment: analogue weighing scale, flexible metallic tape, aneroid sphygmomanometer, stethoscope, and glucometer. A supervised dry-run of the data collection was done on the fourth day.

Patient and Public Engagement

There was no patient involvement. However, participants who needed treatment were referred to nearby community clinic for further evaluation and referral if needed.

Data Collection

The community health workers interviewed subjects, measured height, weight and causal capillary blood glucose. The interview included information on age, sex, education, occupation, tobacco use, treatment for hypertension and diabetes, and history of CVD event. The questionnaire was in Bangla, adapted from the national STEPS survey 2010 [10]. Height and weight were measured for calculating body mass index (kg/m^2). Blood pressure was measured twice using an appropriately sized arm cuff while the participants were in seating position. An aneroid sphygmomanometer (ALRK, Japan) was used. The first measurement was performed after five minutes of rest, and the second measurement was taken two minutes after the first measurement. We used the second measurement values in the analyses. Capillary blood glucose irrespective of prandial status [11] was measured using a glucometer (Accu-Check Softclix CE0088, Roche Diabetes Care, Germany) on the right index finger avoiding much stasis.

Estimation of 10-year CVD risk

We estimated 10-year CVD risk using 2014 WHO/ISH risk prediction charts (hereinafter referred to as CVD risk chart) for South East Asia Region-D without cholesterol [5] for a fatal or non-fatal major cardiovascular event. The variables used for this estimation were age (40–49 years, 50-59 years, 60-69 years, and ≥70 years), sex (men and women), systolic blood pressure (<140, 140-159, 160-179, and ≥180 mmHg), smoking status (smoker or non-smoker), and the presence or absence of diabetes (casual capillary blood glucose >11.1 mmol/L or use of medication for diabetes). The 10-year CVD risk categories were low (<10%), moderate (10 –

For peer review only - http://bmjopen.bmj.com/site/about/guidelines.xhtml

19.9%), high (20-29.9%), and very high (≥ 30%) [5]. Prior to analysis, weighting of the data was done to account for selection probabilities and non-response to remove bias from a survey sample and make the results better project the target population (all citizens of Debhatta aged 40 years or older) [12].

Data Analysis

A total of 1,600 participants (734 were men and 866 women) could be recruited by approaching 1,721 consecutive eligible subjects (response rate, 93.0%). Among them, 55 had a history of CVD events (heart attack or stroke) and were excluded from our analysis as recommended [5]. Finally, our analysis is based on 1545 persons.

Mean (standard deviation) for continuous variables and proportion for categorical variables were obtained. Results on risk categories were presented for sexes combined and separately. Age stratification of the risk categories were not done because it has already been taken in to account for calculating the risk. We categorized the CVD risk into low (<10%), moderate (10 to 19.9%), high (20 to 29.9%), and very high (≥30%) [5]. Complex sample analyses were done to obtain weighted prevalence estimates and 95% confidence intervals (CIs) The 95% CIs of the risk estimates were used to examine differences between groups, overlapping intervals were considered non-significant different at 5% level.

Furthermore, we have estimated the prevalence after adding those with antihypertensive medication having normal blood pressure <140/90 mm Hg to the chart-calculated proportions as per CVD risk chart practice notes for clinicians [5 13]. This approach has ensured that duplicate counts of individuals are not done for those who were classified using systolic blood pressure categories.

RESULTS

Background Characteristics

Results are based on 697 men (45.1%) and 848 women (54.9%) (**Table 1**). Their mean age was 53.9 years, with a standard deviation of 11.6 years. The number of people persistently decreased from (42.4%) in the youngest age group (40-49 years) to 13.9% in the oldest group (70 years or older). Less than one-quarter (24.3%) of them completed primary education. The majority

For peer review only - http://bmjopen.bmj.com/site/about/guidelines.xhtml

(86.1%) of women were housewives, and the most common occupation in men was agriculture and other manual works (45.8%).

Smoking was almost absent in women (1.2%) but common in men (36.6%). Most participants had SBP< 140 mmHg (76.0%), which gradually declined thru intervening categories to 1.6% in the ≥180 mmHg category. More than one-third (36.0%) people had hypertension (blood pressure ≥140/90 mmHg or history of medication), and one in ten (9.4%) had diabetes (capillary blood glucose >11.1 mmol/L or medication).

Distribution of 10-year CVD Risk

All subsequent analyses were weighted. The distribution of 10-year CVD risk in the study participants are presented for sexes combined and separately (**Table 2**). Most participants had a low risk of CVD (81.5%, 95% CI 78.4–84.6), while the moderate, high, and very high risk were 9.9 % (7.4–12.1), 5.8% (4.4–7.2), and 2.8% (1.5–4.1) respectively. In general, women had relatively high prevalence of moderate, high, and very high risk compared to men, albeit statistically non-significant as indicated by overlapping 95% CIs. A sub-group analysis indicated that 10.9% people of low risk group had high systolic blood pressure (≥140 mm Hg). This persistently increased to 97.5% in the very high risk group (**Figure 1**). However, these proportions were relatively small in case of high blood glucose (≥11.1 mmol/L), 4.8% to 31.7% **(Figure 2).** Obesity (body mass index ≥30 kg/m^2) also showed an increasing trend across CVD risk categories (results not shown).

Age-standardized results of very high CVD risk across sex groups are presented in **Table 3**. The prevalence was 2.9%. Men had lower prevalence (1.9%) compared to women (4.4%). We added proportion of people with self-reported medication history for hypertension having controlled blood pressure (<140/90 mm Hg), the overall proportion increased to 8.5%% (6.5% in men and 11.1% in women).

DISCUSSION

The present study is the first-ever population-based study in Bangladesh to estimate the 10-year CVD risk scores done by community health workers. In this household survey of people aged 40 years or older living in a rural area of Bangladesh, we found that 2.9% had very-high CVD risk who require medication for treatment. Addition of history of medication for hypertension (and

For peer review only - http://bmjopen.bmj.com/site/about/guidelines.xhtml

controlled blood pressure, <140/90 mm Hg) increased this to 8.5%. These findings underscore the importance of pharmacological interventions in a cost-effective manner and use of community health workers for CVD prevention and control measures.

The Risk Charts were developed for the LMICs [6], which is pertinent for Bangladeshi population. In a representative sample of an Upazila, we found a low risk for the 10-year CVD risk in majority (81.5%) of the adults aged ≥ 40 years living in a rural area. This estimate is comparable to South-East Asia regional estimate [14], and neighboring Asian countries such as Pakistan (79.2%) [15], Nepal (86.4%) [16], Sri Lanka (86.4%) [17], and India (82.7%) [18]. These, however, should be interpreted with caution because of differences in age of the subject, which is an important determinant. Another point is the version of the Chart. A few previous studies used 2007 version, we used 2014 version. Therefore, our results may not be directly comparable without considering the chart version and age group of the subjects. Three studies in Bangladesh reported CVD risk in adults [19-21]. Two of them were done in rural populations. The proportion of people who had high risk (≥ 20%) varied largely from 2.1% [20] to 10.0% [21] between two studies. Therefore, a conclusive prevalence estimate for rural area is yet to be drawn for Bangladesh. There might be community-specific features to influence the risk estimates.

Out of pocket health care expenditure in Bangladesh is very high (60%), and most of this expenditure is for medicines [22]. We believe that the use of 10-year CVD risk would decrease the out of pocket- and other-costs [15]. The total risk approach may help reducing the patients' load to the health system too, which is already overburdened in Bangladesh. Because the community health workers can detect CVD risk at household level, the coverage of primary health care for the prevention of CVD events would be more efficient if they are used.

Despite a large proportion of deaths due to CVDs in Bangladesh [23], preventive services for CVDs in Bangladesh are limited. Only 16% of health care facilities (i.e., hospitals, community clinics) had the resources to diagnose, treat and manage patients with CVDs [24]. The Upazila health complexes provide both preventive and curative services. Next to the Upazila health complexes the union level health and family welfare centres cater services to people through doctors, medical assistants and family welfare visitors depending on their availability. The ultimate level of health care facilities in rural areas of Bangladesh are the community clinics at the door steps of people. The services at these facilities are limited to the measurement of blood pressure or referrals only [24]. These centres can provide better services if these can be

For peer review only - http://bmjopen.bmj.com/site/about/guidelines.xhtml

integrated into the domiciliary services provided by the community health workers. The current study provides evidences, for the first time, that health workers can screen out potential CVD risk for further evaluation at next level.

Although we provide evidence that community health care workers can contribute to detection of people with a high risk of CVD for initiating treatment at primary health care facilities by physicians, they need to be trained well. Further studies are warranted to study more on the cut-off points (20% or 30%) of CVD risk charts for initiating medicines. Both these cut-off points have potentials of over-and -under-diagnosis. Nonetheless, most studies favor a 30% cut-off point for CVD risk estimation. WHO has recommended that at least 80% of health facilities should have the availability of affordable basic technologies and essential medicines necessary to treat significant NCDs, including CVDs [4]. Screening of rural adults by community health workers could contribute substantially to achieve this target.

The number of people who needs these services based on single risk factor would be huge to deal by a limited number of health service providers where supplies and medicines remain scarce. By integrating the CVD risk prediction charts into the national guidelines for the management of NCDs, the resource needs could be easily reduced without compromising the quality of services to those who are at high risk of CVDs. The implementation of CVD risk prediction chart may become popular if adequate supply of essential medicines is ensured, which is currently inadequate [8], to fixed facilities where they will be referred. The use of local health assistants to conduct the survey at household level, a weighted analysis for the local Upazila and age-standardization for Bangladeshi population at large provided an inherent strength of community participation. Considering the similarity of the environment around the community clinics the current study has a high potential of replication all over Bangladesh.

Our study had limitations too. The estimates that we present here are based on South-East Asian regional criteria of the CVD Risk Charts in the absence of any national criteria. There might be between-country difference in applicability of the prediction charts. Besides, the study was done in a single upazila, not in Bangladesh as a whole. Absence of cholesterol data and use of casual blood glucose data in absence of classic symptoms of diabetes are limitations too. Further, our data were collected back in 2015, and we cannot analyze out data using 2019 CVD risk chart [14] because the control measures were already planned based on our findings. We

For peer review only - http://bmjopen.bmj.com/site/about/guidelines.xhtml

presume that the data of CVD risk using the 2019 charts will be different than what we report here [25].

Conclusion

One in five rural Bangladeshi people aged 40 years or older need treatment for CVD prevention. This information can be used for planning resource for CVD prevention. The use of community health workers to estimate their 10-year CVD risk could be option in Bangladesh. This approach has a potential for better coverage of essential health care for those who are at a very high risk of CVD events. There is a need for national level study covering diversities of rural areas. Future studies should consider including the obesity and total cholesterol in line with 2019 CVD prediction chart.

Footnotes

Ethical considerations

This survey was done by the Upazila Health System to see whether they community workers can get CVD risk factor data and provide feedbacks to the community. In addition, acceptance of household level measurement of blood pressure etc and counselling services provided by the community health workers were examined. Communities were encouraged to participate. Community consents were obtained engaging the management committee of the community clinics, union councils and management committees of all schools before launching the initiative. These were in line with the ethics committee approval of the National Center for Control of Rheumatic Fever and Heart Diseases. The Committee approved getting the consent verbally because this assessment was a health service-oriented work as per the mandate of the Upazila Health Complex. Participants had the rights to withdraw at any stage of the assessment and not to participate at all. Participants were given a report of their risk factor status and referral was done for treatment if necessary. All the health workers of the Upazila irrespective of their involvement in the assessment were trained so that the health benefits reach out all the households of Debhatta Upazila.

Consent to publish: All authors have given consent to the publication of this manuscript.

For peer review only - http://bmjopen.bmj.com/site/about/guidelines.xhtml

Competing Interests: The authors of this study declare no conflict of interest.

Funding: Financial assistance for this study was provided by the WHO Bangladesh (WHO Reference: 2015/545398-0, Purchase Order: 201288322)

Authors contributions: MMZ: conceptualized the project, developed the protocol, trained manpower, analyzed data, interpreted results critically, and drafted the manuscript. MZ, KNC, SZ: prepared the manual and trained the field team, implemented the survey in the field, revised the manuscript, and AHMEH guided the work, coordinated the fieldwork, and reviewed the manuscript critically. He is the guarantor of data. All authors have approved the submission.

Collaborators: Omar Ali (Programme Manager) and Abdul Alim (Deputy Programme Manager) of NCD Control at Directorate General of Health Services, Dhaka; SMA Rahman (UHFPO) of Debhatta Upazila Health Complex, Satkhira; and Alamgir Sikdar (Project Officer), Subrato Gosh (Field Coordinator), and Masud Ahmed (Data Manager) of the WHO-Government joint PEN Project.

Acknowledgments: The authors are grateful to the field team especially the health inspectors of Debhatta Upazila Health Complex for organizing the training of the health assistants and collecting the data. The authors thank Reazwanul Haque Khan and Suraiya Akter of WHO for providing logistic and secretarial support to the PEN project. The health assistants of Debhatta are Md. Waheduj Zaman, Al-hamra Parvin, Debashis Sardar, Munjila Khatun, Md. Ashadul Hoque, Helali Begum, Supria Ghosh, Farhana Sultana, Md Tazibur Rahman, Dipa Rani Adhikary, SK Sharifuzzaman, Md. Abdul Alim, Sree Sipra Rani, SK Rashekul Islam, S M Tazwddin Ahmed, Masuma Yeasmin

Disclaimer: The authors alone are responsible for the views expressed in this article, and they do not necessarily represent the views, decisions, or policies of the institutions with which they are affiliated.

Availability of data and materials: Data are available upon reasonable request. Please contact Dr M. Mostafa Zaman at zamanm@who.int

For peer review only - http://bmjopen.bmj.com/site/about/guidelines.xhtml

Table 1. Characteristics of the study participants, Bangladeshi rural adults aged 40 years or older years, results are number (percent)

Characteristics	Total (n = 1545)	Men (n = 697)	Women (n = 848)
Age in years, mean (SD)	53.9 (11.6)	56.4 (11.7)	51.8 11.2)
Age categories			
40-49	655 (42.4)	229 (32.9)	426 (50.2)
50-59	425 (27.5)	209 30.0	216 (25.4)
60-69	250 (16.2)	130 (18.7)	120 (14.2)
≥70	215 (13.9)	129 (18.5)	86 (10.1)
Education, above primary	375 (24.3)	247 (35.4)	128 (15.1)
Occupation			
Business (small or big)	232 (15.0)	228 (32.4)	4 (0.5)
Agriculture and other manual works	358 (23.2)	322 (45.8)	36 (4.3)
Household works	749 (48.4)	21 (3.0)	728 (86.1)
Others*	207 (13.4)	132 (18.8)	75 (8.9)
Smoking tobacco	265 (17.2)	255 (36.6)	10 (1.2)
Obesity, body mass index ≥30 kg/m^2	155 (7.4)	40 (5.7)	75 (8.8)
Systolic blood pressure categories, mmHg			
<140	1174 (76.0)	571 (81.9)	603 (71.1)
140-159	257 (16.6)	95 (13.6)	162 (19.1)
160-179	85 (5.5)	23 (3.3)	61 (7.3)
≥180	27 (1.8)	6 (0.9)	21 (2.5)
Diastolic blood pressure categories, mmHg			
<90	1109 (71.8)	528 (75.6)	581 (68.5)
90-99	263 (17.2)	113 (16.2)	150 (17.7)
≥100	173 (11.2)	56 (8.0)	117 (13.8)
Medication for			
Hypertension	225 (14.6)	72 (10.3)	153 (18.0)
Diabetes	111 (7.2)	51 (7.3)	60 (7.1)
Hypertension§	556 (36.0)	206 (29.6)	350 (41.3)
Diabetes†	141 (9.1)	65 (9.3)	76 (9.0)

*Includes: salaried work, unemployed, weaver, beggar, cook, tailor, cobbler, etc.

§Blod pressure≥140/90 mmHg, or use of antihypertension medicaton;

†Random capillary glucose ≥11.1 mmol/L, or use of anti-diabetes medication

355

For peer review only

Table 2. Percent (95% confidence interval) people having categories of 10-year CVD risk (fatal and non-fatal)*

CVD risk categories	Men and women (n=1545)	Men (n=697)	Women (n=848)
Low (<10 %)	81.5 (78.4-84.6)	85.0 (80.4-89.6)	78.2 (74.4-81.9)
Moderate (10-19.9%)	9.9 (7.4-12.1)	7.5 (5.2-9.9)	12.1 (9.0-15.2)
High (20-29.9%)	5.8 (4.4-7.2)	5.5 (3.4-7.6)	6.1 (4.5-7.6)
Very high (≥30%)	2.8 (1.5-4.1)	1.9 (0.4-2.5)	3.7 (1.9-5.5)

*Using the WHO/ISH South East Asia Region-D charts without cholesterol;

For peer review only

For peer review only - http://bmjopen.bmj.com/site/about/guidelines.xhtml

Table 3. Age standardized* prevalence (95% confidence interval) of people aged ≥40 years with very high CVD risk ≥30%) using the WHO/ISH Chart plus anti-hypertensive medication¶

CVD risk categories	Men and women (n=1545)	Men (n=697)	Women (n=848)
Simple use of chart	2.9 (0.7-5.2)	1.9 (0.2-4.1)	4.4 (1.0-8.5)
Chart plus medication for hypertension¶	8.5 (5.0-12.0)	6.5 (1.9-11.1)	11.1 (5.8-16.5)

*Standardized for Bangladesh population age structure of Census 2011

¶For only those used medication and had controlled blood pressure (<140/90 mm Hg). Others were already accounted for by systolic blood pressure criteria

357

358

359

For peer review only

For peer review only - http://bmjopen.bmj.com/site/about/guidelines.xhtml

References

1. Kyu HH, Abate D, Abate KH, et al. Global, regional, and national disability-adjusted life-years (DALYs) for 359 diseases and injuries and healthy life expectancy (HALE) for 195 countries and territories, 1990–2017: a systematic analysis for the Global Burden of Disease Study 2017. *The Lancet* 2018;392(10159):1859-922. doi: 10.1016/S0140-6736(18)32335-3
2. World Health Organization. Noncommunicable diseases country profiles 2018. Geneva, Switzerland: World Health Organization, 2018:223.
3. Checkley W, Ghannem H, Irazola V, et al. Management of NCD in low- and middle-income countries. *Glob Heart* 2014;9(4):431-43. doi: 10.1016/j.gheart.2014.11.003
4. World Health Organization. NCD Global Monitoring Framework: Ensuring progress on noncommunicable diseases in countries.: World Health Organization; [Available from: https://www.who.int/nmh/global_monitoring_framework/en/ accessed 30 Jan 2021.
5. World Health Organization. World Health Organization/International Society of Hypertension (WH0/ISH) risk prediction charts for 14 WHO epidemiological sub-regions (charts in colour): World Health Organization, 2007.
6. Mendis S, Lindholm LH, Mancia G, et al. World Health Organization (WHO) and International Society of Hypertension (ISH) risk prediction charts: assessment of cardiovascular risk for prevention and control of cardiovascular disease in low and middle-income countries. *J Hypertens* 2007;25(8):1578-82. doi: 10.1097/HJH.0b013e3282861fd3
7. Riaz BK, Ali L, Ahmad SA, et al. Community clinics in Bangladesh: A unique example of public-private partnership. *Heliyon* 2020;6(5):e03950. doi: 10.1016/j.heliyon.2020.e03950
8. Zaman M, Ullah A, Bhuiyan M, et al. Noncommunicable disease prevention and control situation in a primary health care setting of Bangladesh: design and baseline findings of an intervention. *Chronic Dis Int* 2016;3(1):1-9.
9. National Center for Control of Rheumatic Fever and Heart Diseases Dhaka, Bangladesh: Directorate General of Health Services, Ministry of Health and Family Welfare, Government of the People's Republic of Bangladesh; [Available from: http://nccrfhd.org/ accessed 30 Jan 2021.
10. Zaman MM, Rahman MM, Rahman MR, et al. Prevalence of risk factors for non-communicable diseases in Bangladesh: Results from STEPS survey 2010. *Indian J Public Health* 2016;60(1):17-25. doi: 10.4103/0019-557X.177290 [published Online First: 2016/02/26]
11. World Health Organization. Global report on diabetes. Geneva, Switzerland: World Health Organization 2016.
12. Hakim F, Bhuiyan R, Akter K, Zaman MM. Weighting National Survey Data for Bangladeshi Population. 2020. https://methods.sagepub.com/case/weighting-national-survey-data-for-bangladeshi-population (accessed 30 Jan 2021).
13. Otgontuya D, Oum S, Buckley BS, et al. Assessment of total cardiovascular risk using WHO/ISH risk prediction charts in three low and middle income countries in Asia. *BMC Public Health* 2013;13:539. doi: 10.1186/1471-2458-13-539
14. The WHO CVD Risk Chart Working Group. World Health Organization cardiovascular disease risk charts: revised models to estimate risk in 21 global regions. *The Lancet Glob Health* 2019;7(10):e1332-e45. doi: 10.1016/S2214-109X(19)30318-3
15. Mendis S, Lindholm LH, Anderson SG, et al. Total cardiovascular risk approach to improve efficiency of cardiovascular prevention in resource constrain settings. *J Clin Epidemiol* 2011;64(12):1451-62. doi: 10.1016/j.jclinepi.2011.02.001
16. Khanal MK, Ahmed MS, Moniruzzaman M, et al. Total cardiovascular risk for next 10 years among rural population of Nepal using WHO/ISH risk prediction chart. *BMC Res Notes* 2017;10(1):120. doi: 10.1186/s13104-017-2436-9

For peer review only - http://bmjopen.bmj.com/site/about/guidelines.xhtml

17. Ranawaka UK, Wijekoon CN, Pathmeswaran A, et al. Risk estimates of cardiovascular diseases in a Sri Lankan community. *Ceylon Med J* 2016;61(1):11-7. doi: 10.4038/cmj.v61i1.8253
18. Ghorpade AG, Shrivastava SR, Kar SS, et al. Estimation of the cardiovascular risk using World Health Organization/International Society of Hypertension (WHO/ISH) risk prediction charts in a rural population of South India. *Int J Health Policy Manag* 2015;4(8):531-6. doi: 10.15171/ijhpm.2015.88
19. Ahmed MSAM, Moniruzzaman M, Chowdhury S, et al. Cardiovascular Risk Assessment Among Urban Population of Bangladesh Using WHO/ISH Risk Prediction Chart. *International Journal of Epidemiology* 2015;44(suppl_1):i202-i02. doi: 10.1093/ije/dyv096.327
20. Cravedi P, Sharma SK, Bravo RF, et al. Preventing renal and cardiovascular risk by renal function assessment: insights from a cross-sectional study in low-income countries and the USA. *BMJ Open* 2012;2(5) doi: 10.1136/bmjopen-2012-001357
21. Fatema K, Zwar NA, Milton AH, et al. Cardiovascular risk assessment among rural population: findings from a cohort study in a peripheral region of Bangladesh. *Public Health* 2016;137:73-80. doi: 10.1016/j.puhe.2016.02.016
22. Directorate General of Helath Services. Multisectoral Action Plan for the Noncommunicable Disease Control and Prevention (2016-2021). Dhaka, Bangladesh: Directorate General of Helath Services, Ministry of Health and Family Welfare, Government of the People's Republic Bangladesh., 2015.
23. Bangladesh Bureau of Statistics. Health and Morbidity Status Survey-2012 Dhaka, Bangladesh: Bangladesh Bureau of Statistics, Ministry of Planning, Goverment of the Peoples Republic of Bangladesh 2013 [Available from: http://bbs.portal.gov.bd/sites/default/files/files/bbs.portal.gov.bd/page/4c7eb0f0_e780_4686_b546_b4fa0a8889a5/HMSS.pdf accessed 30 Jan 2021.
24. National Institute of Population Research and Training, Associates for Community and Population Research, and ICF International. Bangladesh Health Facility Survey 2014. Dhaka, Bangladesh: National Institute of Population Research and Training, Associates for Community and Population Research, and ICF International. , 2016.
25. Islam JY, Zaman MM, Moniruzzaman M, et al. Estimation of total cardiovascular risk using the 2019 WHO CVD prediction charts and comparison of population-level costs based on alternative drug therapy guidelines: a population-based study of adults in Bangladesh. *BMJ Open* 2020;10(7):e035842. doi: 10.1136/bmjopen-2019-035842

For peer review only - http://bmjopen.bmj.com/site/about/guidelines.xhtml

Figure Legends

Figure 1. Weighted proportion of people with high blood pressure (≥140/90 mm Hg) across four 10-year CVD risk categories* according to WHO/ISH risk scores (n=1545)

* figure 1 footnotes: Low risk indicates <10%; Moderate risk, 10-19.9%; High risk, 20-29.9%, very high risk, ≥30%; Error bars indicate 95% confidence intervals.

Figure 2. Weighted proportion of people with high blood glucose (≥11.1 mmol/L) across four 10-year CVD risk categories* according to WHO/ISH risk scores (n=1545)

* figure 2 footnotes: Low risk indicates <10%; Moderate risk, 10-19.9%; High risk, 20-29.9%, very high risk, ≥30%; Error bars indicate 95% confidence intervals.

For peer review only

For peer review only - http://bmjopen.bmj.com/site/about/guidelines.xhtml

Figure 1

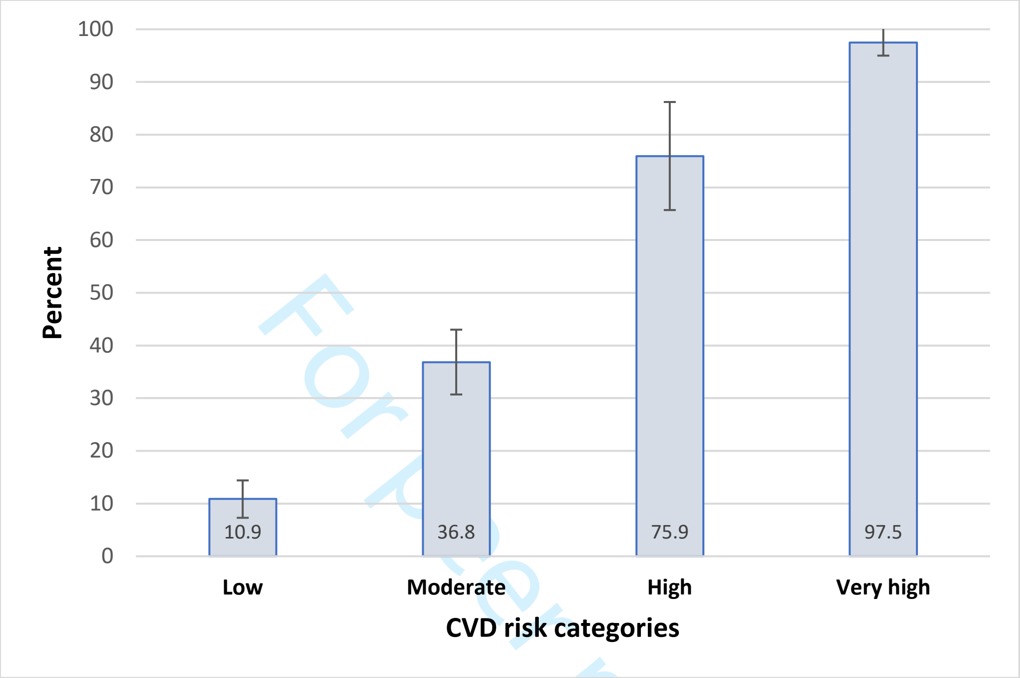

For peer review only

Figure 2

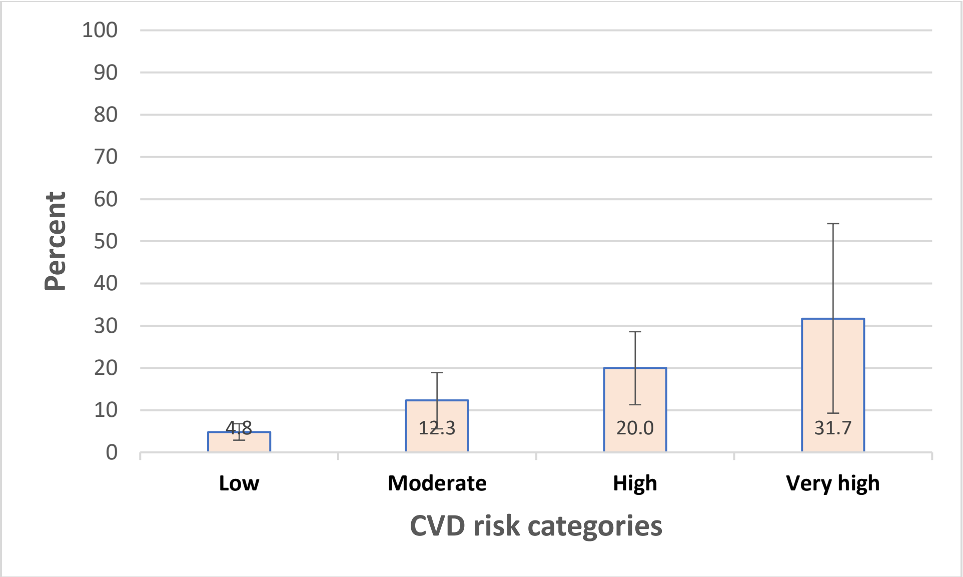
100
90
80
70
60
50
40
30
20
10
0
Percent
4.8
12.3
20.0
31.7
Low
Moderate
High
Very high
CVD risk categories

STROBE Statement—Checklist of items that should be included in reports of ***cross-sectional studies***

	Item No	Recommendation	Page No
**Title and abstract**	1	(*a*) Indicate the study's design with a commonly used term in the title or the abstract	1
		(*b*) Provide in the abstract an informative and balanced summary of what was done and what was found	2
**Introduction**			
Background/rationale	2	Explain the scientific background and rationale for the investigation being reported	4
Objectives	3	State specific objectives, including any prespecified hypotheses	5
**Methods**			
Study design	4	Present key elements of study design early in the paper	5
Setting	5	Describe the setting, locations, and relevant dates, including periods of recruitment, exposure, follow-up, and data collection	5
Participants	6	(*a*) Give the eligibility criteria, and the sources and methods of selection of participants	5
Variables	7	Clearly define all outcomes, exposures, predictors, potential confounders, and effect modifiers. Give diagnostic criteria, if applicable	6
Data sources/ measurement	8*	For each variable of interest, give sources of data and details of methods of assessment (measurement). Describe comparability of assessment methods if there is more than one group	*6-7*
Bias	9	Describe any efforts to address potential sources of bias	6
Study size	10	Explain how the study size was arrived at	4-5
Quantitative variables	11	Explain how quantitative variables were handled in the analyses. If applicable, describe which groupings were chosen and why	6-7
Statistical methods	12	(*a*) Describe all statistical methods, including those used to control for confounding	6-7
		(*b*) Describe any methods used to examine subgroups and interactions	6-7
		(*c*) Explain how missing data were addressed	6
		(*d*) If applicable, describe analytical methods taking account of sampling strategy	na
		(*e*) Describe any sensitivity analyses	na
**Results**			
Participants	13*	(a) Report numbers of individuals at each stage of study—eg numbers potentially eligible, examined for eligibility, confirmed eligible, included in the study, completing follow-up, and analysed	6-7
		(b) Give reasons for non-participation at each stage	6
		(c) Consider use of a flow diagram	na
Descriptive data	14*	(a) Give characteristics of study participants (eg demographic, clinical, social) and information on exposures and potential confounders	7-8
		(b) Indicate number of participants with missing data for each variable of interest	na
Outcome data	15*	Report numbers of outcome events or summary measures	6-7
Main results	16	(*a*) Give unadjusted estimates and, if applicable, confounder-adjusted estimates and their precision (eg, 95% confidence interval). Make clear which confounders were adjusted for and why they were included	7-8

For peer review only

For peer review only - http://bmjopen.bmj.com/site/about/guidelines.xhtml

		(*b*) Report category boundaries when continuous variables were categorized	6
		(*c*) If relevant, consider translating estimates of relative risk into absolute risk for a meaningful time period	na
Other analyses	17	Report other analyses done—eg analyses of subgroups and interactions, and sensitivity analyses	na
**Discussion**			
Key results	18	Summarise key results with reference to study objectives	8
Limitations	19	Discuss limitations of the study, taking into account sources of potential bias or imprecision. Discuss both direction and magnitude of any potential bias	10
Interpretation	20	Give a cautious overall interpretation of results considering objectives, limitations, multiplicity of analyses, results from similar studies, and other relevant evidence	10
Generalisability	21	Discuss the generalisability (external validity) of the study results	10
**Other information**			
Funding	22	Give the source of funding and the role of the funders for the present study and, if applicable, for the original study on which the present article is based	11

*Give information separately for exposed and unexposed groups.

Note: An Explanation and Elaboration article discusses each checklist item and gives methodological background and published examples of transparent reporting. The STROBE checklist is best used in conjunction with this article (freely available on the Web sites of PLoS Medicine at http://www.plosmedicine.org/, Annals of Internal Medicine at http://www.annals.org/, and Epidemiology at http://www.epidem.com/). Information on the STROBE Initiative is available at www.strobe-statement.org.

For peer review only

BMJ Open

Estimated total cardiovascular risk in a rural area of Bangladesh: A household level cross-sectional survey done by local community health workers

Journal:	*BMJ Open*
Manuscript ID	bmjopen-2020-046195.R2
Article Type:	Original research
Date Submitted by the Author:	08-Apr-2021

Complete List of Authors:	Zaman, MM; World Health Organization Regional Office for South-East Asia, WHO Country Office for Bangladesh<br>Moniruzzaman, Mohammad; Shiga University of Medical Science, Public Health<br>Chowdhury, Kamrun ; National Centre for Control of Rheumatic Fever and Heart Diseases, Epidemiology<br>Zareen, Salma; National Centre for Control of Rheumatic Fever and Heart Diseases, Director<br>Hossain, A.H.M. Enayet; Directorate General of Health Services, NonCommunicable Diseases Control Programme

**Primary Subject Heading**:	Cardiovascular medicine
Secondary Subject Heading:	Public health
Keywords:	Cardiac Epidemiology < CARDIOLOGY, PREVENTIVE MEDICINE, PRIMARY CARE, Hypertension < CARDIOLOGY, DIABETES & ENDOCRINOLOGY

SCHOLARONE™
Manuscripts

BMJ

I, the Submitting Author has the right to grant and does grant on behalf of all authors of the Work (as defined in the below author licence), an exclusive licence and/or a non-exclusive licence for contributions from authors who are: i) UK Crown employees; ii) where BMJ has agreed a CC-BY licence shall apply, and/or iii) in accordance with the terms applicable for US Federal Government officers or employees acting as part of their official duties; on a worldwide, perpetual, irrevocable, royalty-free basis to BMJ Publishing Group Ltd ("BMJ") its licensees and where the relevant Journal is co-owned by BMJ to the co-owners of the Journal, to publish the Work in this journal and any other BMJ products and to exploit all rights, as set out in our licence.

The Submitting Author accepts and understands that any supply made under these terms is made by BMJ to the Submitting Author unless you are acting as an employee on behalf of your employer or a postgraduate student of an affiliated institution which is paying any applicable article publishing charge ("APC") for Open Access articles. Where the Submitting Author wishes to make the Work available on an Open Access basis (and intends to pay the relevant APC), the terms of reuse of such Open Access shall be governed by a Creative Commons licence – details of these licences and which Creative Commons licence will apply to this Work are set out in our licence referred to above.

Other than as permitted in any relevant BMJ Author's Self Archiving Policies, I confirm this Work has not been accepted for publication elsewhere, is not being considered for publication elsewhere and does not duplicate material already published. I confirm all authors consent to publication of this Work and authorise the granting of this licence.

For peer review only

Estimated total cardiovascular risk in a rural area of Bangladesh: A household level cross-sectional survey done by local community health workers

Running title: Ten-year CVD risk in a rural community of Bangladesh

Mohammad Mostafa Zaman[1], Mohammad Moniruzzaman [2], Kamrun Nahar Chowdhury[3], Salma Zareen[3], AHM Enayet Hussain[4]

Author Affiliations:
[1]World Health Organization, Country Office for Bangladesh, Dhaka, Bangladesh; email: zamanm@who.int
[2]Center for Epidemiologic Research in Asia, Shiga University of Medical Science, Otsu, Shiga, Japan; email: mmzbd82@gmail.com

[3] National Center for Control of Rheumatic Fever and Heart Diseases, Dhaka, Bangladesh; email: c.kamrun2014@gmail.com, s.zareen@live.com

[4]Directorate General of Health Services, Dhaka, Bangladesh; email: paedeye@yahoo.com

ORCID:
Mohammad Mostafa Zaman (000-0002-1736-1342);

Mohammad Moniruzzaman (0000-0003-2144-7111);

Kamrun Nahar Chowdhury (0000-0001-9465-9655);

Word counts: Abstract: 284; Main text: 2796

Number of tables: 3

Number of figures: 2

Address for correspondence:

Dr M Mostafa Zaman
Adviser Research and Publication
World Health Organization
10 Gulshan Avenue, Dhaka 1212
Bangladesh
Phone: +88 0960 40 27200 (hunting)
Email: zamanm@who.int

For peer review only

Abstract

Objective: The aim of this study was to estimate 10-year cardiovascular disease (CVD) risk among Bangladeshi rural community residents, using the 2014 World Health Organization/International Society of Hypertension (WHO/ISH) Risk Prediction Charts.

Study design: Cross-sectional population-based study done by local community health care workers engaging the lowest level facilities of the primary health care system.

Setting and participants: A total of 1545 rural adults aged ≥40 years of Dehatta Upazila of Satkhira district of Bangladesh participated in this survey done in 2015. The community health workers collected data on age, smoking, blood pressure, blood glucose, and treatment history of diabetes and hypertension.

Primary outcome measures: We estimated total 10-year CVD risk using the WHO/ISH South East Asia Region-D charts without cholesterol and categorized the risk into low (<10%), moderate (10 to 19.9%), high (20 to 29.9%), and very high (≥30%).

Results: The participants' mean age (±SD) was 53.9±11.6 years. Overall, the 10-year CVD risks (%, 95% CI) were as follows: low risk (81.55%, 78.4-84.6), moderate risk (9.9%, 7.4–12.1%), high risk (5.8%, 4.4–7.2%), and very high risk (2.8%, 1.5–4.1%). In women, moderate to very high risks were higher (moderate 12.1%, high 6.1%, and very high 3.7%) compared to men (moderate 7.5%, high 5.5%, and very high 1.9%) but none of these were statistically significant. The age-standardized prevalence of very high risk increased from 2.9% (0.7-5.2%) to 8.5% (5.0-12.0) when those with anti-hypertensive medication having controlled blood pressure (<140/90 mmHg) added.

Conclusion: The very high-risk estimates could be used for planning resource for CVD prevention programme at Upazila level. There is a need for a national level study, covering diversities of rural areas, to contribute to national planning of CVD prevention.

Keywords: Rural community, cardiovascular disease, cardiovascular risk, Bangladesh

For peer review only

For peer review only - http://bmjopen.bmj.com/site/about/guidelines.xhtml

Article Summary

Strengths and limitations of the study

- The present study is the first-ever population-based study in Bangladesh done by community health workers to identify those who need drug therapy CVD prevention (using the 2014 WHO/ISH CVD risk prediction charts) under guidance of the local health authority.
- The use of local community health workers to conduct the survey at household level provided an inherent strength of community participation. Current study has a high potential of replication all over Bangladesh because Upazila health system is similar everywhere.
- The estimates that we present here are based on South-East Asian regional criteria of the CVD Risk Charts in the absence of any national criteria is an inherent weakness of the approach.
- There is threat of generalizability because the survey was done in a single Upazila of a country having hundreds of Upazila.
- Absence of cholesterol data and use of casual blood glucose data in absence of classic symptoms of diabetes are limitations too.

For peer review only - http://bmjopen.bmj.com/site/about/guidelines.xhtml

BACKGROUND

Cardiovascular disease (CVD) is the leading cause of deaths globally, taking an estimated 17.9 million lives in 2016, representing 31% of all deaths [1 2]. Of these deaths, 85% are due to heart attack and stroke. Over three quarters of CVD deaths take place in low-and middle-income countries (LMICs) [3]. The World Health Organization's (WHO) South-East Asia Region has eleven countries, all of which fall under the LMIC category.

Deaths from CVDs are, to a great extent, preventable through targeting their risk factors. These risk factors include smoking, unhealthy diet, alcohol in excess, sedentary behavior, high blood glucose and high blood pressure. Identification and management of individual risk factors need a huge effort from the health system. To help reduce the global burden of CVDs, WHO member states have committed to provide counselling and drug treatments for at least 50% of eligible people (defined as aged 40 years or older and at high risk of CVDs) by 2025 [4]. Accordingly, a total risk approach developed by WHO/ISH makes the approach easier and cost-effective. [5 6]

In Bangladesh, a South-East Asian country, 30% of the total deaths were due to CVD in 2016 [2]. National health system's response to identify population at high risk for CVDs is still absent [2]. The availability of trained health workforce, especially community health workers, is a prerequisite to implementing such programmes. Primary health care system in rural areas of Bangladesh includes fixed facility services (Upazila health complex, union sub-centres and community clinics), and domiciliary services provided by community health workers. The community clinics (total 13,000) are the lowest level and most peripheral facilities catering services to approximately 6,000 people of surrounding area.[7] These clinics are run by community health care providers for catering services for selected communicable diseases[7]. They screen patients for hypertension and diabetes and treat them under the guidance of doctors working in the union sub-centres and upazila health complexes. However, their linkage to the community health workers are not well established.

Our previous study showed that the basic Package for Essential NCD (PEN) intervention for noncommunicable disease (NCD) prevention, especially CVD, is a feasible and realistic option at primary health care level [8]. However, the proportion of rural people who are at high

For peer review only - http://bmjopen.bmj.com/site/about/guidelines.xhtml

risk of CVDs are not known. The community health workers' capacity to contribute to CVD prevention at community level is also unknown. Their engagement in a low resource setting like ours might substantially contribute to primary prevention efforts for screening, counseling and community awareness. Therefore, we aimed at conducting a household level assessment by community health workers to determine the proportion of population at high-risk for CVD in the catchment area of community clinics of a selected upazila in Bangladesh.

METHODS

Study design and setting

Bangladesh has 492 Upazila (as of 19 December 2017), which is the second lowest tier of regional administration in Bangladesh after a district (total 64). One upazila on average has eight unions. Debhata is one of the Upazila of Satkhira district located in south-western part of Bangladesh. It's projected population for 2015 was 132,303. The adults of Debhatta have five years of median schooling [8], which is classical of Bangladeshi rural area. We selected Debhata upazila because the country's first NCD intervention was implemented there in 2013-2014 [8].

Experienced community health workers collected data at household level from August to December 2015 in Debhatta Upazila. Participants were resident adults aged 40 years or older residing in the catchment areas of all 16 community clinics of the Upazila. These 16 community clinics were located in 16 of 57 "Mauza" (the primary sampling units of rural area designated by the statistical authority of Bangladesh). All these community clinics had a list of households in their respective "Mauza". In each Mauza, community health workers selected 100 eligible residents consecutively starting from the first household in the list and continued visiting households until 100 could be selected. Thus, the total sample size was 1600. At least two home visits were done to keep the non-response minimum. As usual, several people were not present at home during data collectors' visit. Refusal was almost absent. Before deployment for the data collection, 16 community health workers from their respective area were trained for three days. They were given a set of equipment: analogue weighing scale, flexible metallic tape, aneroid sphygmomanometer, stethoscope, and glucometer. A supervised dry-run of the data collection was done on the fourth day.

Patient and Public Engagement

For peer review only - http://bmjopen.bmj.com/site/about/guidelines.xhtml

There was no patient involvement. However, participants who needed treatment were referred to nearby community clinic for further evaluation and referral if needed.

Data Collection

The community health workers interviewed subjects, measured height, weight and causal capillary blood glucose. The interview included information on age, sex, education, occupation, tobacco use, treatment for hypertension and diabetes, and history of CVD event. The questionnaire was in Bangla, adapted from the national STEPS survey 2010 [9]. Height and weight were measured for calculating body mass index (kg/m^2). Blood pressure was measured twice using an appropriately sized arm cuff while the participants were in seating position. An aneroid sphygmomanometer (ALRK, Japan) was used. The first measurement was performed after five minutes of rest, and the second measurement was taken two minutes after the first measurement. We used the second measurement values in the analyses. Capillary blood glucose irrespective of prandial status [10] was measured using a glucometer (Accu-Check Softclix CE0088, Roche Diabetes Care, Germany) on the right index finger avoiding much stasis.

Estimation of 10-year CVD risk

We estimated 10-year CVD risk using 2014 WHO/ISH risk prediction charts (hereinafter referred to as CVD risk chart) for South East Asia Region-D without cholesterol [5] for a fatal or non-fatal major cardiovascular event. The variables used for this estimation were age (40–49 years, 50-59 years, 60-69 years, and ≥70 years), sex (men and women), systolic blood pressure (<140, 140-159, 160-179, and ≥180 mmHg), smoking status (smoker or non-smoker), and the presence or absence of diabetes (casual capillary blood glucose >11.1 mmol/L or use of medication for diabetes). The 10-year CVD risk categories were low (<10%), moderate (10 – 19.9%), high (20-29.9%), and very high (≥ 30%) [5]. Prior to analysis, weighting of the data was done to account for selection probabilities and non-response to remove bias from a survey sample and make the results better project the target population (all citizens of Debhatta aged 40 years or older) [11].

Data Analysis

For peer review only - http://bmjopen.bmj.com/site/about/guidelines.xhtml

A total of 1,600 participants (734 were men and 866 women) could be recruited by approaching 1,721 consecutive eligible subjects (response rate, 93.0%). Among them, 55 had a history of CVD events (heart attack or stroke) and were excluded from our analysis as recommended [5]. Finally, our analysis is based on 1545 persons.

Mean (standard deviation) for continuous variables and proportion for categorical variables were obtained. Results on risk categories were presented for sexes combined and separately. Age stratification of the risk categories were not done because it has already been taken in to account for calculating the risk. We categorized the CVD risk into low (<10%), moderate (10 to 19.9%), high (20 to 29.9%), and very high (≥30%) [5]. Complex sample analyses were done to obtain weighted prevalence estimates and 95% confidence intervals (CIs) The 95% CIs of the risk estimates were used to examine differences between groups, overlapping intervals were considered non-significant different at 5% level.

Furthermore, we have estimated the prevalence after adding those with antihypertensive medication having normal blood pressure (<140/90 mm Hg) to the chart-calculated proportions as per CVD risk chart practice notes for clinicians [5 12]. This approach has ensured that duplicate counts of individuals are not done for those who were classified using systolic blood pressure categories.

<u>Ethics approval</u>

We obtained ethical approval for this study from the ethics committee of the National Center for Control of Rheumatic Fever and Heart Diseases [13] (No. NCCRFHD/2015/01). We provided participants with detailed information regarding the study objectives, procedures and how the data will be utilized by the local health authority using a print and verbal means in Bangla. We informed potential participants that they deserve the right not to participate and withdraw from the study at any stage of data collection. Consent was obtained verbally considering a high illiteracy rate in the area as approved by the ethics committee.

RESULTS

<u>Background Characteristics</u>

Results are based on 697 men (45.1%) and 848 women (54.9%) (**Table 1**). Their mean age was 53.9 years, with a standard deviation of 11.6 years. The number of people persistently decreased

For peer review only - http://bmjopen.bmj.com/site/about/guidelines.xhtml

from (42.4%) in the youngest age group (40-49 years) to 13.9% in the oldest group (70 years or older). Less than one-quarter (24.3%) of them completed primary education. The majority (86.1%) of women were housewives, and the most common occupation in men was agriculture and other manual works (45.8%).

Smoking was almost absent in women (1.2%) but common in men (36.6%). Most participants had SBP< 140 mmHg (76.0%), which gradually declined thru intervening categories to 1.6% in the ≥180 mmHg category. More than one-third (36.0%) people had hypertension (blood pressure ≥140/90 mmHg or history of medication), and one in ten (9.4%) had diabetes (capillary blood glucose >11.1 mmol/L or medication).

Distribution of 10-year CVD Risk

All subsequent analyses were weighted. The distribution of 10-year CVD risk in the study participants are presented for sexes combined and separately (**Table 2**). Most participants had a low risk of CVD (81.5%, 95% CI 78.4–84.6), while the moderate, high, and very high risk were 9.9 % (7.4–12.1), 5.8% (4.4–7.2), and 2.8% (1.5–4.1) respectively. In total 18.5% had moderate to very high level of risk. In general, women had relatively high prevalence of moderate, high, and very high risk compared to men, albeit statistically non-significant as indicated by overlapping 95% CIs.

A sub-group analysis indicated that 10.9% people of low risk group had high systolic blood pressure (≥140 mm Hg). This persistently increased to 97.5% in the very high risk group (**Figure 1**). However, these proportions were relatively small in case of high blood glucose (≥11.1 mmol/L), 4.8% to 31.7% **(Figure 1).** Obesity (body mass index ≥30 kg/m^2) also showed an increasing trend across CVD risk categories (results not shown).

Age-standardized results of very high CVD risk across sex groups are presented in **Table 3**. The prevalence was 2.9%. Men had lower prevalence (1.9%) compared to women (4.4%). We added proportion of people with self-reported medication history for hypertension having controlled blood pressure (<140/90 mm Hg), the overall proportion increased to 8.5%% (6.5% in men and 11.1% in women).

DISCUSSION

For peer review only - http://bmjopen.bmj.com/site/about/guidelines.xhtml

The present study is the first-ever population-based study in Bangladesh to estimate the 10-year CVD risk scores done by community health workers. In this household survey of people aged 40 years or older living in a rural area of Bangladesh, we found that 2.9% had very-high CVD risk who require medication for treatment. Addition of history of medication for hypertension (and controlled blood pressure, <140/90 mm Hg) increased this to 8.5%. These findings underscore the importance of pharmacological interventions in a cost-effective manner and use of community health workers for CVD prevention and control measures.

The Risk Charts were developed for the LMICs [6], which is pertinent for Bangladeshi population. In a representative sample of an Upazila, we found a low risk for the 10-year CVD risk in majority (81.5%) of the adults aged ≥ 40 years living in a rural area. This estimate is comparable to South-East Asia regional estimate [14], and neighboring Asian countries such as Pakistan (79.2%) [15], Nepal (86.4%) [16], Sri Lanka (86.4%) [17], and India (82.7%) [18]. These, however, should be interpreted with caution because of differences in age of the subject, which is an important determinant. Another point is the version of the Chart. A few previous studies used 2007 version, we used 2014 version. Therefore, our results may not be directly comparable without considering the chart version and age group of the subjects. Three studies in Bangladesh reported CVD risk in adults [19-21]. Two of them were done in rural populations. The proportion of people who had high risk (≥ 20%) varied largely from 2.1% [20] to 10.0% [21] between two studies. Therefore, a conclusive prevalence estimate for rural area is yet to be drawn for Bangladesh. There might be community-specific features to influence the risk estimates.

Out of pocket health care expenditure in Bangladesh is very high (60%), and most of this expenditure is for medicines [22]. We believe that the use of 10-year CVD risk would decrease the out of pocket- and other-costs [15] although this has a potential of under-treatment. The total risk approach may help reducing the patients' load to the health system too, which is already overburdened in Bangladesh. Because the community health workers can detect CVD risk at household level, the coverage of primary health care for the prevention of CVD events would be more efficient if they are used.

Despite a large proportion of deaths due to CVDs in Bangladesh [23], preventive services for CVDs in Bangladesh are limited. Only 16% of health care facilities (i.e., hospitals, community clinics) had the resources to diagnose, treat and manage patients with CVDs [24]. The Upazila health complexes provide both preventive and curative services. Next to the Upazila

For peer review only - http://bmjopen.bmj.com/site/about/guidelines.xhtml

health complexes the union level health and family welfare centres cater services to people through doctors, medical assistants and family welfare visitors depending on their availability. The ultimate level of health care facilities in rural areas of Bangladesh are the community clinics at the door steps of people. The services at these facilities are limited to the measurement of blood pressure or referrals only [24]. These centres can provide better services if these can be integrated into the domiciliary services provided by the community health workers. The current study provides evidences, for the first time, that health workers can screen out potential CVD risk for further evaluation at next level. Considering relative complexities of the CVD risk estimation methods, hypertension detection and treatment at community level could be an entry point because 97.5% of those who must get treatment (very high risk group) have hypertension. Three-quarter (75.9%) of the high risk group also can be brought under treatment using blood pressure screening at community.

Although we provide evidence that community health care workers can contribute to detection of people with a high risk of CVD for initiating treatment at primary health care facilities by physicians, they need to be trained well. Further studies are warranted to study more on the cut-off points (20% or 30%) of CVD risk charts for initiating medicines. Both these cut-off points have potentials of over-and -under-diagnosis. Nonetheless, most studies favor a 30% cut-off point for CVD risk estimation. WHO has recommended that at least 80% of health facilities should have the availability of affordable basic technologies and essential medicines necessary to treat significant NCDs, including CVDs [4]. Screening of rural adults by community health workers could contribute substantially to achieve this target.

The number of people who needs these services based on single risk factor would be huge to deal by a limited number of health service providers where supplies and medicines remain scarce. By integrating the CVD risk prediction charts into the national guidelines for the management of NCDs, the resource needs could be easily reduced without compromising the quality of services to those who are at high risk of CVDs. The implementation of CVD risk prediction chart may become popular if adequate supply of essential medicines is ensured, which is currently inadequate [8], to fixed facilities where they will be referred. The use of local health assistants to conduct the survey at household level, a weighted analysis for the local Upazila and age-standardization for Bangladeshi population at large provided an inherent strength of

For peer review only - http://bmjopen.bmj.com/site/about/guidelines.xhtml

community participation. Considering the similarity of the environment around the community clinics the current study has a high potential of replication all over Bangladesh.

Our study had limitations too. The estimates that we present here are based on South-East Asian regional criteria of the CVD Risk Charts in the absence of any national criteria. There might be between-country difference in applicability of the prediction charts. Besides, the study was done in a single upazila, not in Bangladesh as a whole. Absence of cholesterol data and use of casual blood glucose data in absence of classic symptoms of diabetes are limitations too. Further, our data were collected back in 2015, and we cannot analyze out data using 2019 CVD risk chart [14] because the control measures were already planned based on our findings. We presume that the data of CVD risk using the 2019 charts will be different than what we report here [25].

Conclusion

One in five rural Bangladeshi people aged 40 years or older need treatment for CVD prevention. Despite having a potential of under-treatment, this information can be used for planning resource for CVD prevention considering resource constraints. The use of community health workers to estimate their 10-year CVD risk could be option in Bangladesh. This approach has a potential for better coverage of essential health care for those who are at a very high risk of CVD events. There is a need for national level study covering diversities of rural areas. Future studies should consider including the obesity and total cholesterol in line with 2019 CVD prediction chart.

Footnotes

Ethical considerations

This survey was done by the Upazila Health System to see whether they community workers can get CVD risk factor data and provide feedbacks to the community. In addition, acceptance of household level measurement of blood pressure etc and counselling services provided by the community health workers were examined. Communities were encouraged to participate. Community consents were obtained engaging the management committee of the community clinics, union councils and management committees of all schools before launching the initiative. These were in line with the ethics committee approval of the National Center for Control of Rheumatic Fever and Heart Diseases. The Committee approved getting the consent verbally

For peer review only - http://bmjopen.bmj.com/site/about/guidelines.xhtml

because this assessment was a health service-oriented work as per the mandate of the Upazila Health Complex. Participants had the rights to withdraw at any stage of the assessment and not to participate at all. Participants were given a report of their risk factor status and referral was done for treatment if necessary. All the health workers of the Upazila irrespective of their involvement in the assessment were trained so that the health benefits reach out all the households of Debhatta Upazila.

Consent to publish: All authors have given consent to the publication of this manuscript.

Competing Interests: The authors of this study declare no conflict of interest.

Funding: Financial assistance for this study was provided by the WHO Bangladesh (WHO Reference: 2015/545398-0, Purchase Order: 201288322)

Authors contributions: MMZ: conceptualized the project, developed the protocol, trained manpower, analyzed data, interpreted results critically, and drafted the manuscript. MZ, KNC, SZ: prepared the manual and trained the field team, implemented the survey in the field, revised the manuscript, and AHMEH guided the work, coordinated the fieldwork, and reviewed the manuscript critically. He is the guarantor of data. All authors have approved the submission.

Collaborators: Omar Ali (Programme Manager) and Abdul Alim (Deputy Programme Manager) of NCD Control at Directorate General of Health Services, Dhaka; SMA Rahman (UHFPO) of Debhatta Upazila Health Complex, Satkhira; and Alamgir Sikdar (Project Officer), Subrato Gosh (Field Coordinator), and Masud Ahmed (Data Manager) of the WHO-Government joint PEN Project.

Acknowledgments: The authors are grateful to the field team especially the health inspectors of Debhatta Upazila Health Complex for organizing the training of the health assistants and collecting the data. The authors thank Reazwanul Haque Khan and Suraiya Akter of WHO for providing logistic and secretarial support to the PEN project. The health assistants of Debhatta are Md. Waheduj Zaman, Al-hamra Parvin, Debashis Sardar, Munjila Khatun, Md. Ashadul Hoque, Helali Begum, Supria Ghosh, Farhana Sultana, Md Tazibur Rahman, Dipa Rani

For peer review only - http://bmjopen.bmj.com/site/about/guidelines.xhtml

Adhikary, SK Sharifuzzaman, Md. Abdul Alim, Sree Sipra Rani, SK Rashekul Islam, S M Tazwddin Ahmed, Masuma Yeasmin

Disclaimer: The authors alone are responsible for the views expressed in this article, and they do not necessarily represent the views, decisions, or policies of the institutions with which they are affiliated.

Availability of data and materials: Data are available upon reasonable request. Please contact Dr M. Mostafa Zaman at zamanm@who.int

For peer review only

For peer review only - http://bmjopen.bmj.com/site/about/guidelines.xhtml

Table 1. Characteristics of the study participants, Bangladeshi rural adults aged 40 years or older years, results are number (percent)

**Characteristics**	**Total (n = 1545)**	**Men (n = 697)**	**Women (n = 848)**
Age in years, mean (SD)	53.9 (11.6)	56.4 (11.7)	51.8 11.2)
Age categories			
40-49	655 (42.4)	229 (32.9)	426 (50.2)
50-59	425 (27.5)	209 30.0	216 (25.4)
60-69	250 (16.2)	130 (18.7)	120 (14.2)
≥70	215 (13.9)	129 (18.5)	86 (10.1)
Education, above primary	375 (24.3)	247 (35.4)	128 (15.1)
Occupation			
Business (small or big)	232 (15.0)	228 (32.4)	4 (0.5)
Agriculture and other manual works	358 (23.2)	322 (45.8)	36 (4.3)
Household works	749 (48.4)	21 (3.0)	728 (86.1)
Others*	207 (13.4)	132 (18.8)	75 (8.9)
Smoking tobacco	265 (17.2)	255 (36.6)	10 (1.2)
Obesity, body mass index ≥30 kg/m^2	155 (7.4)	40 (5.7)	75 (8.8)
Systolic blood pressure categories, mmHg			
<140	1174 (76.0)	571 (81.9)	603 (71.1)
140-159	257 (16.6)	95 (13.6)	162 (19.1)
160-179	85 (5.5)	23 (3.3)	61 (7.3)
≥180	27 (1.8)	6 (0.9)	21 (2.5)
Diastolic blood pressure categories, mmHg			
<90	1109 (71.8)	528 (75.6)	581 (68.5)
90-99	263 (17.2)	113 (16.2)	150 (17.7)
≥100	173 (11.2)	56 (8.0)	117 (13.8)
Medication for			
Hypertension	225 (14.6)	72 (10.3)	153 (18.0)
Diabetes	111 (7.2)	51 (7.3)	60 (7.1)
Hypertension[§]	556 (36.0)	206 (29.6)	350 (41.3)
Diabetes†	141 (9.1)	65 (9.3)	76 (9.0)

*Includes: salaried work, unemployed, weaver, beggar, cook, tailor, cobbler, etc.

[§]Blod pressure≥140/90 mmHg, or use of antihypertension medicaton;

†Random capillary glucose ≥11.1 mmol/L, or use of anti-diabetes medication

For peer review only

For peer review only - http://bmjopen.bmj.com/site/about/guidelines.xhtml

Table 2. Percent (95% confidence interval) people having categories of 10-year CVD risk (fatal and non-fatal)*

CVD risk categories	Men and women (n=1545)	Men (n=697)	Women (n=848)
Low (<10 %)	81.5 (78.4-84.6)	85.0 (80.4-89.6)	78.2 (74.4-81.9)
Moderate (10-19.9%)	9.9 (7.4-12.1)	7.5 (5.2-9.9)	12.1 (9.0-15.2)
High (20-29.9%)	5.8 (4.4-7.2)	5.5 (3.4-7.6)	6.1 (4.5-7.6)
Very high (≥30%)	2.8 (1.5-4.1)	1.9 (0.4-2.5)	3.7 (1.9-5.5)

*Using the WHO/ISH South East Asia Region-D charts without cholesterol;

For peer review only

For peer review only - http://bmjopen.bmj.com/site/about/guidelines.xhtml

Table 3. Age standardized* prevalence (95% confidence interval) of people aged ≥40 years with very high CVD risk ≥30%) using the WHO/ISH Chart plus anti-hypertensive medication¶

CVD risk categories	Men and women (n=1545)	Men (n=697)	Women (n=848)
Simple use of chart	2.9 (0.7-5.2)	1.9 (0.2-4.1)	4.4 (1.0-8.5)
Chart plus medication for hypertension¶	8.5 (5.0-12.0)	6.5 (1.9-11.1)	11.1 (5.8-16.5)

*Standardized for Bangladesh population age structure of Census 2011

¶For only those used medication and had controlled blood pressure (<140/90 mm Hg). Others were already accounted for by systolic blood pressure criteria

For peer review only

For peer review only - http://bmjopen.bmj.com/site/about/guidelines.xhtml

References

1. Kyu HH, Abate D, Abate KH, et al. Global, regional, and national disability-adjusted life-years (DALYs) for 359 diseases and injuries and healthy life expectancy (HALE) for 195 countries and territories, 1990–2017: a systematic analysis for the Global Burden of Disease Study 2017. *The Lancet* 2018;392(10159):1859-922. doi: 10.1016/S0140-6736(18)32335-3
2. World Health Organization. Noncommunicable diseases country profiles 2018. Geneva, Switzerland: World Health Organization, 2018:223.
3. Checkley W, Ghannem H, Irazola V, et al. Management of NCD in low- and middle-income countries. *Glob Heart* 2014;9(4):431-43. doi: 10.1016/j.gheart.2014.11.003
4. World Health Organization. NCD Global Monitoring Framework: Ensuring progress on noncommunicable diseases in countries.: World Health Organization; [Available from: https://www.who.int/nmh/global_monitoring_framework/en/ accessed 30 Jan 2021.
5. World Health Organization. World Health Organization/International Society of Hypertension (WH0/ISH) risk prediction charts for 14 WHO epidemiological sub-regions (charts in colour): World Health Organization, 2007.
6. Mendis S, Lindholm LH, Mancia G, et al. World Health Organization (WHO) and International Society of Hypertension (ISH) risk prediction charts: assessment of cardiovascular risk for prevention and control of cardiovascular disease in low and middle-income countries. *J Hypertens* 2007;25(8):1578-82. doi: 10.1097/HJH.0b013e3282861fd3
7. Riaz BK, Ali L, Ahmad SA, et al. Community clinics in Bangladesh: A unique example of public-private partnership. *Heliyon* 2020;6(5):e03950. doi: 10.1016/j.heliyon.2020.e03950
8. Zaman M, Ullah A, Bhuiyan M, et al. Noncommunicable disease prevention and control situation in a primary health care setting of Bangladesh: design and baseline findings of an intervention. *Chronic Dis Int* 2016;3(1):1-9.
9. Zaman MM, Rahman MM, Rahman MR, et al. Prevalence of risk factors for non-communicable diseases in Bangladesh: Results from STEPS survey 2010. *Indian J Public Health* 2016;60(1):17-25. doi: 10.4103/0019-557X.177290 [published Online First: 2016/02/26]
10. World Health Organization. Global report on diabetes. Geneva, Switzerland: World Health Organization 2016.
11. Hakim F, Bhuiyan R, Akter K, Zaman MM. Weighting National Survey Data for Bangladeshi Population. 2020. https://methods.sagepub.com/case/weighting-national-survey-data-for-bangladeshi-population (accessed 30 Jan 2021).
12. Otgontuya D, Oum S, Buckley BS, et al. Assessment of total cardiovascular risk using WHO/ISH risk prediction charts in three low and middle income countries in Asia. *BMC Public Health* 2013;13:539. doi: 10.1186/1471-2458-13-539
13. National Center for Control of Rheumatic Fever and Heart Diseases Dhaka, Bangladesh: Directorate General of Health Services, Ministry of Health and Family Welfare, Government of the People's Republic of Bangladesh; [Available from: http://nccrfhd.org/ accessed 30 Jan 2021.
14. The WHO CVD Risk Chart Working Group. World Health Organization cardiovascular disease risk charts: revised models to estimate risk in 21 global regions. *The Lancet Glob Health* 2019;7(10):e1332-e45. doi: 10.1016/S2214-109X(19)30318-3
15. Mendis S, Lindholm LH, Anderson SG, et al. Total cardiovascular risk approach to improve efficiency of cardiovascular prevention in resource constrain settings. *J Clin Epidemiol* 2011;64(12):1451-62. doi: 10.1016/j.jclinepi.2011.02.001
16. Khanal MK, Ahmed MS, Moniruzzaman M, et al. Total cardiovascular risk for next 10 years among rural population of Nepal using WHO/ISH risk prediction chart. *BMC Res Notes* 2017;10(1):120. doi: 10.1186/s13104-017-2436-9

For peer review only - http://bmjopen.bmj.com/site/about/guidelines.xhtml

17. Ranawaka UK, Wijekoon CN, Pathmeswaran A, et al. Risk estimates of cardiovascular diseases in a Sri Lankan community. *Ceylon Med J* 2016;61(1):11-7. doi: 10.4038/cmj.v61i1.8253
18. Ghorpade AG, Shrivastava SR, Kar SS, et al. Estimation of the cardiovascular risk using World Health Organization/International Society of Hypertension (WHO/ISH) risk prediction charts in a rural population of South India. *Int J Health Policy Manag* 2015;4(8):531-6. doi: 10.15171/ijhpm.2015.88
19. Ahmed MSAM, Moniruzzaman M, Chowdhury S, et al. Cardiovascular Risk Assessment Among Urban Population of Bangladesh Using WHO/ISH Risk Prediction Chart. *International Journal of Epidemiology* 2015;44(suppl_1):i202-i02. doi: 10.1093/ije/dyv096.327
20. Cravedi P, Sharma SK, Bravo RF, et al. Preventing renal and cardiovascular risk by renal function assessment: insights from a cross-sectional study in low-income countries and the USA. *BMJ Open* 2012;2(5) doi: 10.1136/bmjopen-2012-001357
21. Fatema K, Zwar NA, Milton AH, et al. Cardiovascular risk assessment among rural population: findings from a cohort study in a peripheral region of Bangladesh. *Public Health* 2016;137:73-80. doi: 10.1016/j.puhe.2016.02.016
22. Directorate General of Helath Services. Multisectoral Action Plan for the Noncommunicable Disease Control and Prevention (2016-2021). Dhaka, Bangladesh: Directorate General of Helath Services, Ministry of Health and Family Welfare, Government of the People's Republic Bangladesh., 2015.
23. Bangladesh Bureau of Statistics. Health and Morbidity Status Survey-2012 Dhaka, Bangladesh: Bangladesh Bureau of Statistics, Ministry of Planning, Goverment of the Peoples Republic of Bangladesh 2013 [Available from: http://bbs.portal.gov.bd/sites/default/files/files/bbs.portal.gov.bd/page/4c7eb0f0_e780_4686_b546_b4fa0a8889a5/HMSS.pdf accessed 30 Jan 2021.
24. National Institute of Population Research and Training, Associates for Community and Population Research, and ICF International. Bangladesh Health Facility Survey 2014. Dhaka, Bangladesh: National Institute of Population Research and Training, Associates for Community and Population Research, and ICF International. , 2016.
25. Islam JY, Zaman MM, Moniruzzaman M, et al. Estimation of total cardiovascular risk using the 2019 WHO CVD prediction charts and comparison of population-level costs based on alternative drug therapy guidelines: a population-based study of adults in Bangladesh. *BMJ Open* 2020;10(7):e035842. doi: 10.1136/bmjopen-2019-035842

For peer review only - http://bmjopen.bmj.com/site/about/guidelines.xhtml

Figure Legends

Figure 1. Weighted proportion of people with high systolic blood pressure (≥140 mm Hg) and high blood glucose (≥11.1 mmol/L) across four 10-year CVD risk categories* according to WHO/ISH risk scores (n=1545)

* figure 1 footnotes: Low risk indicates <10%; Moderate risk, 10-19.9%; High risk, 20-29.9%, very high risk, ≥30%; Error bars indicate 95% confidence intervals.

For peer review only

Figure 1

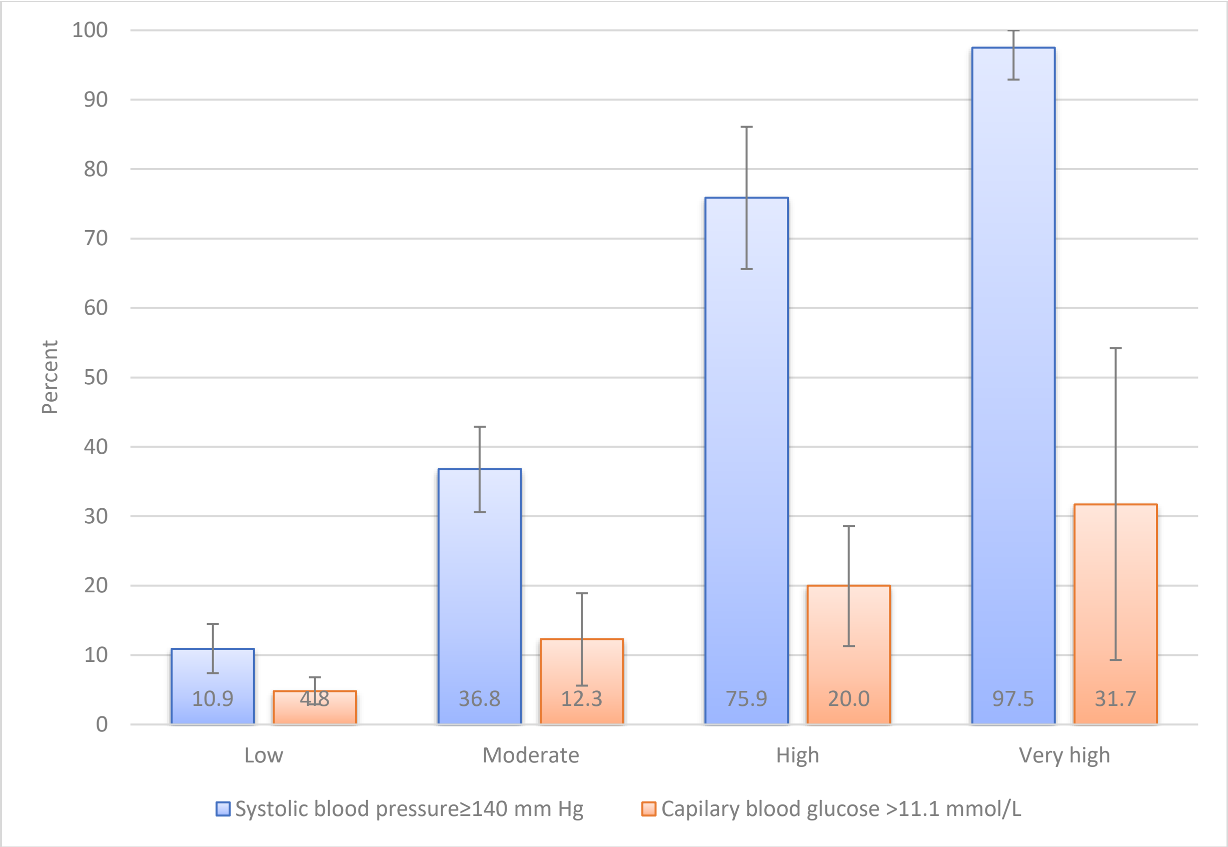
100
90
80
70
60
50
40
30
20
10
0
Percent
10.9
4.8
36.8
12.3
75.9
20.0
97.5
31.7
Low
Moderate
High
Very high
Systolic blood pressure≥140 mm Hg
Capilary blood glucose >11.1 mmol/L

STROBE Statement—Checklist of items that should be included in reports of ***cross-sectional studies***

	Item No	Recommendation	Page No
**Title and abstract**	1	(*a*) Indicate the study's design with a commonly used term in the title or the abstract	1
		(*b*) Provide in the abstract an informative and balanced summary of what was done and what was found	2
**Introduction**			
Background/rationale	2	Explain the scientific background and rationale for the investigation being reported	4
Objectives	3	State specific objectives, including any prespecified hypotheses	5
**Methods**			
Study design	4	Present key elements of study design early in the paper	5
Setting	5	Describe the setting, locations, and relevant dates, including periods of recruitment, exposure, follow-up, and data collection	5
Participants	6	(*a*) Give the eligibility criteria, and the sources and methods of selection of participants	5
Variables	7	Clearly define all outcomes, exposures, predictors, potential confounders, and effect modifiers. Give diagnostic criteria, if applicable	6
Data sources/ measurement	8*	For each variable of interest, give sources of data and details of methods of assessment (measurement). Describe comparability of assessment methods if there is more than one group	*6-7*
Bias	9	Describe any efforts to address potential sources of bias	6
Study size	10	Explain how the study size was arrived at	4-5
Quantitative variables	11	Explain how quantitative variables were handled in the analyses. If applicable, describe which groupings were chosen and why	6-7
Statistical methods	12	(*a*) Describe all statistical methods, including those used to control for confounding	6-7
		(*b*) Describe any methods used to examine subgroups and interactions	6-7
		(*c*) Explain how missing data were addressed	6
		(*d*) If applicable, describe analytical methods taking account of sampling strategy	na
		(*e*) Describe any sensitivity analyses	na
**Results**			
Participants	13*	(a) Report numbers of individuals at each stage of study—eg numbers potentially eligible, examined for eligibility, confirmed eligible, included in the study, completing follow-up, and analysed	6-7
		(b) Give reasons for non-participation at each stage	6
		(c) Consider use of a flow diagram	na
Descriptive data	14*	(a) Give characteristics of study participants (eg demographic, clinical, social) and information on exposures and potential confounders	7-8
		(b) Indicate number of participants with missing data for each variable of interest	na
Outcome data	15*	Report numbers of outcome events or summary measures	6-7
Main results	16	(*a*) Give unadjusted estimates and, if applicable, confounder-adjusted estimates and their precision (eg, 95% confidence interval). Make clear which confounders were adjusted for and why they were included	7-8

For peer review only - http://bmjopen.bmj.com/site/about/guidelines.xhtml

For peer review only

		(*b*) Report category boundaries when continuous variables were categorized	6
		(*c*) If relevant, consider translating estimates of relative risk into absolute risk for a meaningful time period	na
Other analyses	17	Report other analyses done—eg analyses of subgroups and interactions, and sensitivity analyses	na
**Discussion**			
Key results	18	Summarise key results with reference to study objectives	8
Limitations	19	Discuss limitations of the study, taking into account sources of potential bias or imprecision. Discuss both direction and magnitude of any potential bias	10
Interpretation	20	Give a cautious overall interpretation of results considering objectives, limitations, multiplicity of analyses, results from similar studies, and other relevant evidence	10
Generalisability	21	Discuss the generalisability (external validity) of the study results	10
**Other information**			
Funding	22	Give the source of funding and the role of the funders for the present study and, if applicable, for the original study on which the present article is based	11

*Give information separately for exposed and unexposed groups.

Note: An Explanation and Elaboration article discusses each checklist item and gives methodological background and published examples of transparent reporting. The STROBE checklist is best used in conjunction with this article (freely available on the Web sites of PLoS Medicine at http://www.plosmedicine.org/, Annals of Internal Medicine at http://www.annals.org/, and Epidemiology at http://www.epidem.com/). Information on the STROBE Initiative is available at www.strobe-statement.org.

For peer review only
